# Aging promotes accumulation of senescent and multiciliated cells in human endometrial epithelium

**DOI:** 10.1093/hropen/hoae048

**Published:** 2024-08-12

**Authors:** Marina Loid, Darina Obukhova, Keiu Kask, Apostol Apostolov, Alvin Meltsov, Demis Tserpelis, Arthur van den Wijngaard, Signe Altmäe, Galina Yahubyan, Vesselin Baev, Merli Saare, Maire Peters, Ave Minajeva, Priit Adler, Ganesh Acharya, Kaarel Krjutškov, Maria Nikolova, Felipe Vilella, Carlos Simon, Masoud Zamani Esteki, Andres Salumets

**Affiliations:** Department of Obstetrics and Gynecology, Institute of Clinical Medicine, University of Tartu, Tartu, Estonia; Competence Centre on Health Technologies, Tartu, Estonia; Department of Obstetrics and Gynecology, Institute of Clinical Medicine, University of Tartu, Tartu, Estonia; Department of Clinical Genetics, Maastricht University Medical Centre+, Maastricht, The Netherlands; Department of Genetics and Cell Biology, GROW School for Oncology and Developmental Biology, Maastricht University, Maastricht, The Netherlands; Department of Obstetrics and Gynecology, Institute of Clinical Medicine, University of Tartu, Tartu, Estonia; Competence Centre on Health Technologies, Tartu, Estonia; Department of Obstetrics and Gynecology, Institute of Clinical Medicine, University of Tartu, Tartu, Estonia; Competence Centre on Health Technologies, Tartu, Estonia; Competence Centre on Health Technologies, Tartu, Estonia; Department of Clinical Genetics, Maastricht University Medical Centre+, Maastricht, The Netherlands; Department of Genetics and Cell Biology, GROW School for Oncology and Developmental Biology, Maastricht University, Maastricht, The Netherlands; Department of Clinical Genetics, Maastricht University Medical Centre+, Maastricht, The Netherlands; Department of Clinical Genetics, Maastricht University Medical Centre+, Maastricht, The Netherlands; Department of Biochemistry and Molecular Biology, Faculty of Sciences, University of Granada, Granada, Spain; Instituto de Investigación Biosanitaria ibs.GRANADA, Granada, Spain; Division of Obstetrics and Gynecology, Department of Clinical Science, Intervention and Technology, Karolinska Institutet and Karolinska University Hospital, Stockholm, Sweden; Department of Molecular Biology, Faculty of Biology, University of Plovdiv, Plovdiv, Bulgaria; Department of Molecular Biology, Faculty of Biology, University of Plovdiv, Plovdiv, Bulgaria; Department of Obstetrics and Gynecology, Institute of Clinical Medicine, University of Tartu, Tartu, Estonia; Competence Centre on Health Technologies, Tartu, Estonia; Department of Obstetrics and Gynecology, Institute of Clinical Medicine, University of Tartu, Tartu, Estonia; Competence Centre on Health Technologies, Tartu, Estonia; Institute of Biomedicine and Translational Medicine, University of Tartu, Tartu, Estonia; Faculty of Science and Technology, Institute of Computer Science, University of Tartu, Tartu, Estonia; Division of Obstetrics and Gynecology, Department of Clinical Science, Intervention and Technology, Karolinska Institutet and Karolinska University Hospital, Stockholm, Sweden; Department of Obstetrics and Gynecology, Institute of Clinical Medicine, University of Tartu, Tartu, Estonia; Competence Centre on Health Technologies, Tartu, Estonia; Department of Molecular Biology, Faculty of Biology, University of Plovdiv, Plovdiv, Bulgaria; Center for Women’s Health, Plovdiv, Bulgaria; Research & Medical Department, Carlos Simon Foundation, INCLIVA Health Research Institute, Valencia, Spain; Research & Medical Department, Carlos Simon Foundation, INCLIVA Health Research Institute, Valencia, Spain; Beth Israel Deaconess Medical Center, Harvard University, Boston, MA, USA; Department of Pediatrics, Obstetrics & Gynecology, University of Valencia, Valencia, Spain; Department of Obstetrics and Gynecology, Baylor College of Medicine, Houston, TX, USA; Department of Clinical Genetics, Maastricht University Medical Centre+, Maastricht, The Netherlands; Department of Genetics and Cell Biology, GROW School for Oncology and Developmental Biology, Maastricht University, Maastricht, The Netherlands; Division of Obstetrics and Gynecology, Department of Clinical Science, Intervention and Technology, Karolinska Institutet and Karolinska University Hospital, Stockholm, Sweden; Department of Obstetrics and Gynecology, Institute of Clinical Medicine, University of Tartu, Tartu, Estonia; Competence Centre on Health Technologies, Tartu, Estonia; Division of Obstetrics and Gynecology, Department of Clinical Science, Intervention and Technology, Karolinska Institutet and Karolinska University Hospital, Stockholm, Sweden

**Keywords:** aging, endometrium, gene expression, endometrial receptivity, immunohistochemistry, assisted reproduction

## Abstract

**STUDY QUESTION:**

What changes occur in the endometrium during aging, and do they impact fertility?

**SUMMARY ANSWER:**

Both the transcriptome and cellular composition of endometrial samples from women of advanced maternal age (AMA) are significantly different from that of samples from young women, suggesting specific changes in epithelial cells that may affect endometrial receptivity.

**WHAT IS KNOWN ALREADY:**

Aging is associated with the accumulation of senescent cells in aging tissues. Reproductive aging is mostly attributed to the decline in ovarian reserve and oocyte quality, whereas the endometrium is a unique complex tissue that is monthly renewed under hormonal regulation. Several clinical studies have reported lower implantation and pregnancy rates in oocyte recipients of AMA during IVF. Molecular studies have indicated the presence of specific mutations within the epithelial cells of AMA endometrium, along with altered gene expression of bulk endometrial tissue.

**STUDY DESIGN, SIZE, DURATION:**

Endometrial transcriptome profiling was performed for 44 women undergoing HRT during the assessment of endometrial receptivity before IVF. Patients younger than 28 years were considered as the young maternal age (YMA) group (age 23–27 years) and women older than 45 years were considered as the AMA group (age 47–50 years). Endometrial biopsies were obtained on Day 5 of progesterone treatment and RNA was extracted. All endometrial samples were evaluated as being receptive based on the expression of 68 common endometrial receptivity markers. Endometrial samples from another 24 women classified into four age groups (YMA, intermediate age group 1 (IMA1, age 29–35), intermediate age group 2 (IMA2, age 36–44), and AMA) were obtained in the mid-secretory stage of a natural cycle (NC) and used for validation studies across the reproductive lifespan.

**PARTICIPANTS/MATERIALS, SETTING, METHODS:**

A total of 24 HRT samples (12 YMA and 12 AMA) were subject to RNA sequencing (RNA-seq) and differential gene expression analysis, 20 samples (10 YMA and 10 AMA) were used for qPCR validation, and 24 NC samples (6 YMA, 6 IMA1, 6 IMA2 and 6AMA) were used for RNA-seq validation of AMA genes across the woman’s reproductive lifespan. Immunohistochemistry (IHC) was used to confirm some expression changes at the protein level. Computational deconvolution using six endometrial cell type-specific transcriptomic profiles was conducted to compare the cellular composition between the groups.

**MAIN RESULTS AND THE ROLE OF CHANCE:**

Comparisons between YMA and AMA samples identified a lower proportion of receptive endometria in the AMA group (*P* = 0.007). Gene expression profiling identified 491 differentially expressed age-sensitive genes (*P* adj < 0.05) that revealed the effects of age on endometrial epithelial growth and receptivity, likely contributing to decreased reproductive performance. Our results indicate that changes in the expression of the cellular senescence marker p16^INK4a^ and genes associated with metabolism, inflammation, and hormone response are involved in endometrial aging. Importantly, we demonstrate that the proportion of multi-ciliated cells, as discovered based on RNA-seq data deconvolution and tissue IHC results, is affected by endometrial aging, and propose a putative onset of age-related changes. Furthermore, we propose that aging has an impact on the transcriptomic profile of endometrial tissue in the context of endometrial receptivity.

**LARGE SCALE DATA:**

The raw sequencing data reported in this article are deposited at the Gene Expression Omnibus under accession code GSE236128.

**LIMITATIONS, REASONS FOR CAUTION:**

This retrospective study identified changes in the endometrium of patients undergoing hormonal replacement and validated these changes using samples obtained during a NC. However, future studies must clarify the importance of these findings on the clinical outcomes of assisted reproduction.

**WIDER IMPLICATIONS OF THE FINDINGS:**

The findings reported in this study have important implications for devising future strategies aimed at improving fertility management in women of advanced reproductive age.

**STUDY FUNDING/COMPETING INTEREST(S):**

This research was funded by the Estonian Research Council (grant no. PRG1076), Horizon 2020 innovation grant (ERIN, grant no. EU952516), Enterprise Estonia (grant no. EU48695), MSCA-RISE-2020 project TRENDO (grant no. 101008193), EU 874867 project HUTER, the Horizon Europe NESTOR grant (grant no. 101120075) of the European Commission, the EVA specialty program (grant no. KP111513) of the Maastricht University Medical Center (MUMC+), MICIU/AEI/10.13039/501100011033 and FEDER, EU projects Endo-Map (grant no. PID2021-12728OB-100), ROSY (grant no. CNS2022-135999), and the National Science Fund of Bulgaria (grant no. KII-06 H31/2). The authors declare no competing interests.

WHAT DOES THIS MEAN FOR PATIENTS? The twenties are considered the optimal age for female reproduction. However, in the modern era, many women delay their parenting plans, resulting in increased numbers of women seeking infertility treatment later in life. Although assisted reproduction techniques (ARTs), such as IVF, may alleviate the negative impact of advanced maternal age on oocyte quality, the likelihood of conception in the forties is still relatively low. While aging has been linked to endometrial health causing abnormal endometrial thickness, an increased inflammatory background, and altered hormonal responses, we still lack a full understanding of the actual changes in the endometrium on the molecular and cellular level, and how they impact embryo implantation. To explain the age-specific changes, we analysed the gene expression differences in endometrial samples between women of young and advanced ages undergoing HRT and compared the cell type composition of the samples between the two age groups. Our results introduce new knowledge that cellular aging processes may play an important role in the development and functioning of endometrial tissue, and these must be addressed when designing ART protocols for women of advanced reproductive age. Our findings also suggest that particular type of epithelial cells may have potential diagnostic significance for age-related changes. The observed very high proportions of ciliated cells (cells with slender hair-like projections on their surfaces) in some advanced-age patients emphasise the need for further clinical studies to estimate the optimal abundance of ciliated cells in the endometrium for implantation. It is also crucial to investigate whether an increased presence of these ciliated cells hinders embryo implantation or increases reproductive health risks, and therefore requires clinical attention.

## Introduction

Nowadays, women often postpone having children due to socioeconomic reasons. In 2021, the mean maternal age in Europe was slightly above 31 years (European Statistical System, 2022). The fertility rate decreases substantially for women in their late thirties due to a decline in ovarian reserves, which may lead them to seek ARTs, such as IVF. For instance, IVF with donor oocytes has made procreation possible for women wishing to have children at older ages (Lambalk, 2022), and preimplantation genetic testing (PGT) of IVF embryos for age-associated genome aberrations has improved the chances of ART success when older women are using their own oocytes (Sanders *et al.*, 2021).

However, advanced maternal age (AMA) remains a prevailing negative factor in the implantation rate for many women undergoing ART. This limitation mainly arises from the general fertility decline beginning at about the age of 35, which refers to the progressive decline of the ovarian follicle pool, accompanied by subsequent hormonal changes that affect cyclic endometrial maturation. The age-associated decline in ovarian function and altered hormonal regulation of adipose tissue may produce abnormally short or long menstrual cycles, resulting in either thin or hyperplastic endometria, both of which are associated with poor implantation rates (Djerassi *et al.*, 1995; Burger *et al.*, 1999; Onogi *et al.*, 2020).

Early studies analysing women undergoing oocyte donation IVF cycles, in which HRT, also referred as artificial cycle, is often required for recipients, have provided controversial reports on the effect of patient age on implantation rate (Flamigni *et al.*, 1993; Navot *et al.*, 1994; Cano *et al.*, 1995; Abdalla *et al.*, 1997). Later studies using large sample sizes have found that the implantation rate drops significantly for women in their forties, even when using donor oocytes from young women (Toner *et al.*, 2002; Soares *et al.*, 2005; Sanders *et al.*, 2021). Although nowadays, PGT for aneuploidies (PGT-A) prior to embryo transfer can prevent many implantation failures (Sacchi *et al.*, 2019; Sanders *et al.*, 2021), we hypothesized that eliminating genetically abnormal embryos via PGT alone is not optimal for ART in women of AMA, and there are specific age-related molecular processes in the endometrium that need to be taken into account. One study showing higher pregnancy rates in ART cycles with younger gestational carriers undergoing embryo transfer with and without PGT-A, compared to older participants (Perkins *et al.*, 2016), supports our hypothesis. Moreover, genetic studies suggest that age increases the gene mutation burden in endometrial tissue, particularly in epithelial cells (Moore *et al.*, 2020), which could potentially alter endometrial function with respect to receptivity and implantation in both natural and assisted conception, and increase the future risk of uterine neoplasms.

Our previous studies have shown that not only can gene expression modulate the receptivity of endometrial tissue, the proportion of different cell types in the endometrium can also affect the embryo–endometrial dialogue during implantation (Suhorutshenko *et al.*, 2018). As characterizing cell types using different molecular techniques can be challenging due to costly and laborious cell-type isolation techniques or single-cell omics technologies, we introduced computational deconvolution as an accurate and cost-effective method to decompose endometrial tissue into individual cellular fractions (Suhorutshenko *et al.*, 2018). In this study, we examine the molecular and cellular changes that occur in endometrial tissue as women age. To understand the extent of these changes, we compared the transcriptome landscape and tissue cellular composition between women of AMA (47–50 years old) and young maternal age (YMA, 20–27 years old) to estimate the effect of aging on endometrial regeneration and receptivity and validated these changes using endometrial samples from natural cycles (NC) across women’s reproductive lifespan (20–50 years old).

## Materials and methods

### Ethics statement, patients, and samples

Our research complies with all relevant ethical regulations. The research was approved by the Ethics Committee of the University of Tartu (Ethics Approval No. 340-12) and the Ethics Committee of the Faculty of Biology, Plovdiv University (Ethics Approval No. 3/02.09.2019). Informed consent was provided by all participants.

To investigate the association between endometrial receptivity and a woman’s age, we performed the comparison of 1249 anonymized transcriptomic profiles obtained from women undergoing HRT during the assessment of endometrial receptivity with the beREADY algorithm (Meltsov *et al.*, 2023). The profiles were categorized according to their classified receptivity status and compared between age groups of patients aged 20–29, 30–39, 40–44, and 45 and over. To test whether the proportion of patients with any of the classified receptivity statuses changes linearly with age, we used the Chi-squared test for trend in proportions using the *prop.trend.test()* function in R.

**Figure 1.**
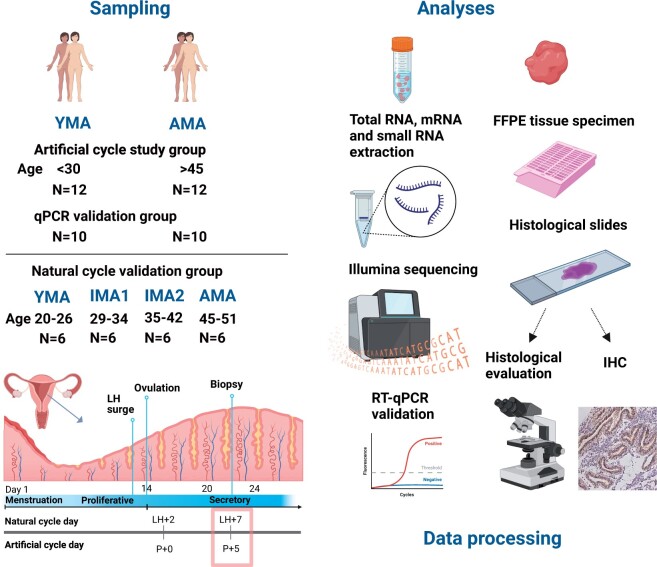
**Schematic overview of the study design and workflow of the analysis.** Endometrial biopsy samples were collected from 22 young maternal age (YMA) and 22 advanced maternal age (AMA) women on Day 5 of progesterone administration (P + 5, artificial cycle) undergoing endometrial receptivity testing using 68 transcriptomic markers and the beREADY algorithm. The study samples underwent mRNA sequencing (12 YMA and 12 AMA samples) and small RNA sequencing (11 YMA and 11 AMA samples), and validation samples (10 YMA and 10 AMA) were used for qPCR validation. Additionally, 24 women donated endometrial samples in the natural cycle. For the analyses, FFPE tissue blocks were prepared for all study samples and haematoxylin and eosin-stained slides were evaluated by the pathologist. Immunohistochemistry (IHC) analysis for p16^INK4a^, C2CD4B, AcTubA, and LhS28 proteins was performed in endometrial samples of both age groups. Sequencing data processing included quality control, adaptor trimming, and alignment to the human genome. Aligned reads were counted and analysed for differential expression of genes between the YMA and AMA groups. Computational deconvolution analysis was performed using single-cell mRNA sequencing data from six endometrial cell populations. The figure was created with BioRender.com.

For whole-transcriptome sequencing, endometrial biopsies were obtained from 44 anonymized women with an assessed endometrial receptivity status in HRT cycles, with all samples classified as receptive by the beREADY classifier. The study group consisted of 12 women aged 20–27 years in the YMA group, 12 women aged 47–50 years in the AMA group, and a validation group consisting of 10 YMA and 10 AMA samples. All women were infertility patients planning frozen embryo transfer (FET) in a HRT cycle following the study biopsy. The only inclusion criterion was the absence of reported uterine pathologies. In HRT, progesterone was administered orally, vaginally, or by injection. The first day of progesterone administration was considered Day 0 (P + 0). An endometrial biopsy for endometrial receptivity testing was taken on Day 5 of progesterone administration (P + 5). All endometrial tissue biopsies were collected using a Pipelle catheter (Laboratoire CCD, Paris, France) and immediately placed into RNAlater (Life Technologies, Waltham, MA, USA). An overview of the study design is presented in Fig. 1. All experiments were performed with blinding of their age group affiliation.

### mRNA sequencing of endometrial samples

#### Library preparation, sequencing, and initial data processing

Total RNA was extracted from all samples using the miRNeasy kit (Qiagen, Hilden, Germany), followed by DNase treatment with DNase Free (Life Technologies), according to the manufacturer’s protocol. RNA integrity was assessed with a Bioanalyzer (Agilent Technologies, Santa Clara, CA, USA) and concentration was measured with a Qubit RNA BR Assay kit (Thermo Fisher, Carlsbad, CA, USA). The cDNA libraries were randomly assigned to two sequencing batches and prepared using the TruSeq Stranded mRNA Sample Preparation Guide (Illumina, San Diego, CA, USA). The quality was assessed using a Bioanalyzer (Agilent). The libraries were pooled and single-read 75 bp sequencing was performed on NextSeq500 (Illumina).

Reads were then mapped to the human reference genome (UCSC release GRCh37/hg19), and quality control and alignment statistics were assessed using MultiQC (v. 1.12). The mapped reads were estimated at the gene level using the Ensembl database for annotation. The batch effect of the sequencing run was taken into account and differentially expressed genes (DEGs) between AMA and YMA groups were identified with a significance criterion of Benjamin and Hochberg adjusted *P*-value < 0.05, where the receptivity score was used as a covariate. The top 20 most significant genes, sorted by log_2_FC, were clustered hierarchically and plotted in a dendrogram. All bioinformatic analysis were performed in R. A detailed description of bioinformatic analyses is presented in Supplementary File S1.

Following the differential expression (DE) analyses, a power estimation for RNA sequencing (RNA-seq) experiments was used to determine the appropriate sample size required to achieve the desired level of statistical power. Using the PROspective Power Evaluation for RNA-seq (PROPER) R package (Wu *et al.*, 2015), it was computed that given the sample size of 12, the significance level (*P* adj) of 0.05, and the effect size (log_2_FC) of 0.3, the power estimate was 85%, whereas for the genes with log_2_FC of at least 1, the power was 99%. Thus, the current study design was considered eligible, with well-powered sample sizes available of 12 YMA and 12 AMA women in the study groups.

To validate the results of RNA-seq analysis, total RNA was extracted from the endometrial samples of the validation group (YMA n = 10, AMA n = 10), then DNA depleted, and cDNA was synthesized using a Maxima First Strand cDNA synthesis kit (Thermo Fisher Scientific Baltics, Vilnius, Lithuania). qRT-PCR experiments were performed in duplicate as previously described (Suhorutshenko *et al.*, 2018). Six loci (*ALDH3A1*, *SPAG6*, *STC1*, *PPP1R1B*, *EML5*, and *C2CD4B*) were selected based on their fold change between the groups and mean expression value in endometrial samples. Oligonucleotide primers are presented in Supplementary Table S1. Expression values were normalized to the endogenous control genes *TBP* and *GAPDH*. An unpaired two-sample Wilcoxon test was applied to estimate the significance level in R.

#### Functional analysis of DEGs

Enrichment analyses for gene ontology (GO) terms and biological pathways were conducted using g: Profiler (v. 0.2.1, updated 19 April 2023; Reimand *et al.*, 2007) and Ingenuity pathway analysis (IPA, Qiagen) tools using all significant DEGs identified between the AMA and YMA groups. For graphical representation of functional analysis data, a GO chord plot was generated using the *GOplot* (v. 1.0.2) package in R. Networks of significant DEGs were then algorithmically generated based on their connectivity in IPA.

The DEGs were then compared with a set of endometrial receptivity genes differentially expressed in the window of implantation (WOI) of healthy women, as reported in previous publications involving biopsies from natural (Hu *et al.*, 2014; Sigurgeirsson *et al.*, 2017; Suhorutshenko *et al.*, 2018) or HRT (Altmäe *et al.*, 2016) cycles. The boxplots were generated with *ggplot* (v. 3.4.0). To explore the role of AMA DEGs in endometrial aging, the DEGs were compared with the lists of genes presented in the GenAge (de Magalhães and Toussaint, 2004), GTExAge (Ren and Kuan, 2020), and the Aging Atlas (Aging Atlas Consortium *et al.*, 2021).

### miRNA sequencing and analysis

For miRNA expression analysis, the miRNeasy advanced micro kit (Qiagen) was used to extract total RNA from the endometrial biopsies. The quality and quantity of the isolated RNA were assessed using RNA TapeStation (Agilent). For library preparation, 100 ng of total RNA was utilized, and subsequent libraries were generated using the NEXTflex Small RNA-seq Library Prep Kit v4 (Perkin Elmer, Waltham, MA, USA). The FASTQ files underwent quality assessment and adapter trimming using FastQC and Trim Galore. The miRGalaxy tool within Galaxy was employed to identify and classify isomiRs, i.e. miRNA sequences that have variations with respect to the reference sequence, based on their read offset relative to the reference miRs (RefSeq) and read copy number (Glogovitis *et al.*, 2021). The IsoRead tool was used to identify miRs and isomiRs, followed by their classification into template and non-template isomiRs using the same module. For DE analysis, count matrices of miRs and isomiRs were analysed using DESeq2, and the expression threshold log2FC ≤ 0.5 and *P*-value < 0.05 were applied. The mRNA targets of miR/isomiRs were predicted using the miRDB database (Chen and Wang, 2020).

### Validation using the NC samples of young, intermediate, and advanced age groups

To investigate whether expression changes found in the AMA group evolve over time, we performed coding RNA-seq of endometrial samples collected from individuals aged between 21 and 51 years. The patients were divided into four age groups: YMA (age 21–27, n = 6), intermediate maternal age group 1 (IMA1; age 28–36, n = 6), intermediate maternal age group 2 (IMA2; 37–44, n = 6) and AMA (45–51, n = 6). Endometrial biopsies were collected during the mid-secretory phase in NC, where no hormonal treatment was used for at least 3 months prior to sampling. There were no significant differences in blood levels of oestradiol and progesterone between the groups. mRNA was extracted from endometrial samples using the miRNeasy micro kit (Qiagen) with on-column DNase treatment (Qiagen), following the manufacturer’s instructions. RNA integrity and concentration were assessed with Qubit (Thermo Fisher) and sequencing libraries were synthesized using a TruSeq Exome RNA Library preparation kit (Illumina). Library quality and molarity were assessed using a TapeStation HS D1000 assay and sequencing was performed on a NextSeq 1000 platform (Illumina).

After the sequencing, read quality and alignment were processed as previously described. Genes with raw count values <4 in over 75% of samples were excluded from the analysis. To compare DEGs among four age groups in a pairwise manner, we used ‘∼Age_Group’ as the value for the ‘design’ argument in the *DESeqDataSetFromMatrix()* function. A detailed description of the analysis is presented in Supplementary File S1.

### Deconvolution

Two single-cell transcriptome datasets of endometrial biopsies, the C1 dataset (library preparation method: Fluidigm; with 2148 cells analysed) and the 10× Genomics dataset (library preparation method: 10× Chromium Next GEM; with 71 032 cells analysed), and their respective cell-type labels were obtained from NCBI’s Gene Expression Omnibus (accession code GSE111976) (Wang *et al.*, 2020). The dataset was chosen based on the criteria of completeness, annotation, sample size, and availability of supportive information. For the menstrual cycle phase, concordance with the bulk RNA-seq samples, single-cell data from the mid-secretory phase, cycle days 20–24 were only included. This resulted in 546-cell and 46 878-cell count matrices for the C1 and 10× Genomics datasets, respectively. These matrices of distinct endometrial cell types were used to calculate the proportions of each cell type in the bulk endometrial samples of the study group, using the dampened weighted least squares (DWLS) method from the *DWLS* R package (v. 0.1.0). To compute the significance of differences in the proportions of each endometrial cell type between the YMA and AMA samples, a non-parametric Mann–Whitney *U*-test (two sides) was used, and Bonferroni correction was applied. To visualize whether the proportion of ciliated epithelial cells correlated with a woman’s age, the samples were ordered according to age. Violin, bar, and scatter plots were produced using the R package *ggplot2* (v. 3.4.0).

### Histological evaluation and immunohistochemistry

For histological analysis of the study group samples, 24 microscope slides with tissue sections (4 μm, three sections per slide) were prepared at the Pathology Department of Tartu University Hospital (Tartu, Estonia) from formalin-fixed paraffin-embedded (FFPE) endometrial tissue sections using a standard haematoxylin and eosin staining protocol (Fischer *et al.*, 2008). The slides were scanned at East Tallinn Central Hospital, using a 3DHistech Panoramic Flash III 250 scanner (3DHistech, Budapest, Hungary) at 20× magnification. The evaluation of cyclic endometrial changes was performed according to Noyes criteria (Noyes *et al.*, 1950) by a pathologist.

To evaluate the expression of cellular senescence protein p16^INK4a^ (p16, CDKN2A), immunohistochemistry (IHC) analysis was performed, using anti-p16^INK4a^, mouse monoclonal antibody against human CDKN2A (ready-to-use solution, Master Diagnostica, Valencia, Spain). Four samples from both the YMA and AMA groups were selected at random, with all samples considered receptive according to the beREADY algorithm. To ensure antibody specificity, tonsil tissue was used as a positive control for p16 staining, and healthy cervix tissue was used as a negative tissue control. FFPE tissue sections on slides were processed according to a standard protocol using the 3,3'-diaminobenzidine (DAB)-based Master Polymer Plus Detection System IHC Kit (Master Diagnostica). Incubation with the primary antibody was carried out overnight in a humidity chamber at 4°C. Further steps were carried out according to the manufacturer’s protocol. The chromogenic reaction was developed for 30 s and stopped thereafter. Cell nuclei were counterstained with Mayer’s haematoxylin solution for 15 s, and then the slides were washed for 10 min with tap water. The slides were dehydrated through graded ethanol and xylene solutions and mounted with a Leica CV mount (Leica Biosystems, Wetzlar, Germany).

For ciliated cell visualization, IHC staining of cilia basal bodies was performed as previously described using a mouse monoclonal anti-LhS28 antibody (Santa Cruz Biotechnology, Heidelberg, Germany) and anti-acetylated tubulin-α (AcTubA; Santa Cruz) was used to stain motile cilia. As a positive control for LhS28 staining, an additional endometrial biopsy was taken on Day 15 of the natural menstrual cycle from a fertile young woman aged 28 years. A fallopian tube biopsy from a 42-year-old woman was used as a positive control for AcTubA. For all negative controls, the primary antibody was omitted from the incubation. All information on the antibodies used in this study is listed in Supplementary Table S1.

Slides were scanned using a Leica SCN 400 slide scanner (Leica Biosystems) with a maximum magnification objective of 20× for IHC analysis and 40× for basal body (BB) and multiciliated cells (MCC) counting. Semi-quantitative analysis of three different areas of scanned sections was conducted with the ImageJ Fiji package (v. 1.52e) (Schindelin *et al.*, 2012). Briefly, the intensity of the DAB signal was measured separately for the stromal, glandular epithelium, and surface epithelium of the endometrium. The relative DAB intensity was calculated using the formula: f = 255 − i, where ‘f’ is relative DAB intensity and ‘i’ is mean DAB intensity obtained from the software ranging from 0 (deep brown, highest expression) to 255 (total white). The Wilcoxon Mann–Whitney test was performed to determine statistical significance. Prism 6 (GraphPad Software, La Jolla, CA, USA) and ImageJ software (Fiji package) were used for data quantification and analysis. BB and MCC counting were performed with blinding by two researchers using anti-LhS28-stained and anti-AcTubA-stained endometrial tissue slides, respectively. The average amounts of ciliated cells exhibiting positive staining between the two measurements were estimated and divided by the area of luminal epithelium (LE). An unpaired sample Student’s *t*-test was applied to test the statistical significance of the differences between the two groups.

### Identification of cilia-associated genes among AMA DEGs

To understand the impact of endometrial aging on cilial cells’ structure and function, we first compared 491 AMA DEGs with the 535 genes and proteins presented in the Human Protein Atlas (https://www.proteinatlas.org), which are highly expressed in the ciliated cells of human fallopian tube and endometrial tissue. The intersecting genes were then searched manually in PubMed (https://pubmed.ncbi.nlm.nih.gov) for evidence of their associations with cilia structures and functions. The expression of cilia-associated AMA DEGs was then analysed pairwise among four age groups.

## Results

### Receptivity of AMA endometrium based on transcriptomic markers

**Figure 2.**
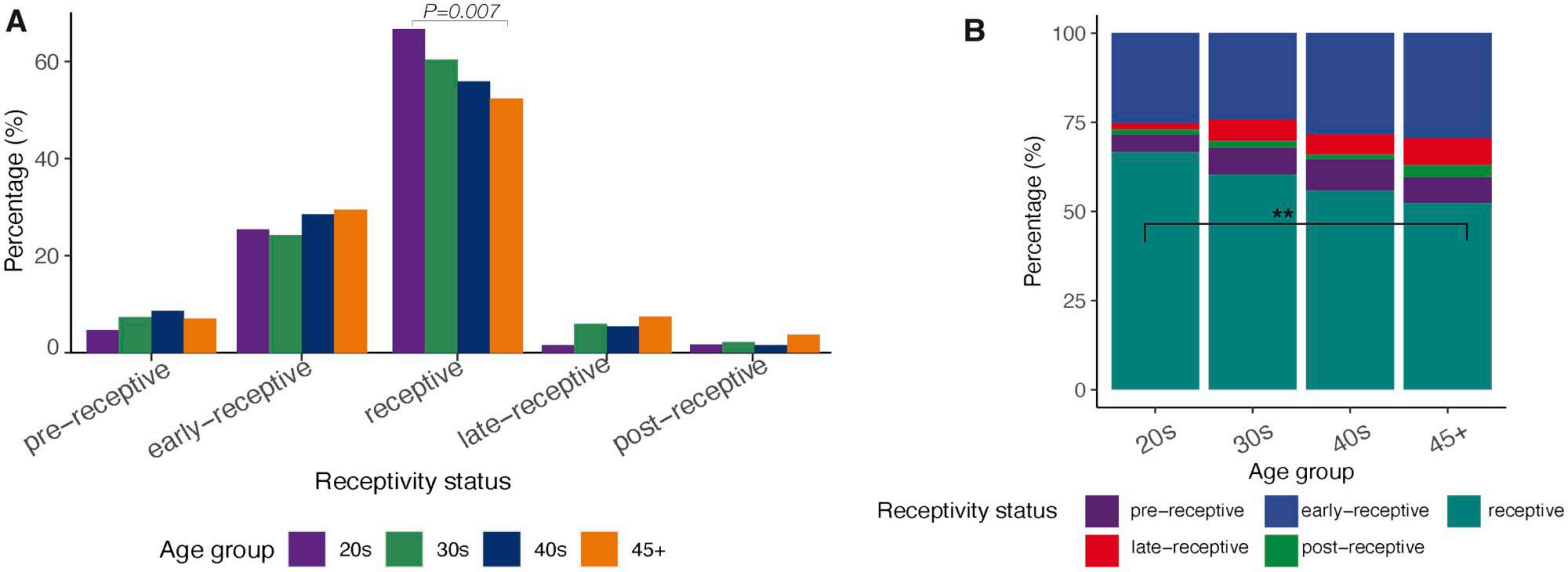
**The results of the analysis of 1249 endometrial receptivity reports based on the beREADY algorithm.** (**A**) The percentage of samples classified as ‘pre-receptive’, ‘early receptive’, ‘receptive’, ‘late receptive’, and ‘post-receptive’ in four age groups, arranged by receptivity status. The proportion of ‘receptive’ samples was significantly lower in women older than 45 years, compared to women aged 20–29 years. Chi-squared test *P*-value = 0.007. (**B**) The proportions of receptivity status, arranged by age. ** Chi-squared test *P*-value < 0.01.

To determine whether a woman’s age may be a factor that affects endometrial receptivity, we compared 1249 endometrial receptivity test reports. We identified that a significantly (*P* = 0.007) lower proportion of samples tested as ‘receptive’ in women over 45 years old compared to women younger than 30 years. The proportions of receptivity status are presented in Fig. 2. The patient group over 45 years exhibited slightly higher proportions of all results of receptivity status, except for receptive result (Fig. 2A), and these changes occurred gradually over the age groups (Fig. 2B).

### DEGs and their respective altered pathways

To analyse the differences in gene expression between the endometria of YMA and AMA women, total RNA samples from the endometrial tissue of 12 young and 12 older women were sequenced. The main characteristics of the study groups are presented in Table 1. In total, 19 357 mRNA transcripts were expressed in the analysed endometrial samples. Based on the gene expression profile, the study samples in each group clustered together. The dendrogram of all study group samples based on the top 50 DEGs is presented in Supplementary Fig. S1A.

**Table 1.** Overview of the study participants.

	YMA	AMA	*P*-value*
	Mean (±SD)	Mean (±SD)	
Study group (N)	12	12	
Age	25.2 (1.2)	47.9 (1.2)	–
BMI	23.9 (4.1)	22.9 (3.1)	ns
Cycle type	HRT: P + 5	HRT: P + 5	
qPCR validation group (N)	10	10	
Age	25.4 (5.1)	47.7 (3.7)	–
BMI	24.1 (5.1)	23.3 (3.8)	ns
Cycle type	HRT: P + 5	HRT: P + 5	

	YMA	IMA1	IMA2	AMA	*P*-value*
	Mean (±SD)	Mean (±SD)	Mean (±SD)	Mean (±SD)	
RNA-seq validation group (N)	6	6	6	6	
Age	22.8 (1.8)	32.2 (1.7)	37.7 (2.8)	47.3 (2.1)	–
BMI	21.6 (1.6)	22.3 (3.1)	24.4 (3.5)	23.8 (2.9)	ns
Cycle type	NC: LH + 7	NC: LH + 7	NC: LH + 7	NC: LH + 7	

YMA, young maternal age group; AMA, advanced maternal age group; IMA1 and IMA2, intermediate maternal age groups 1 and 2, respectively; N, number of participants in each group; HRT, HRT in artificial menstrual cycle; P + 5, Day 5 of progesterone administration; NC, natural menstrual cycle; LH, LH surge day; ns, non-significant.

* Unpaired Student *t*-test *P*-value for pairwise comparisons.

**Figure 3.**
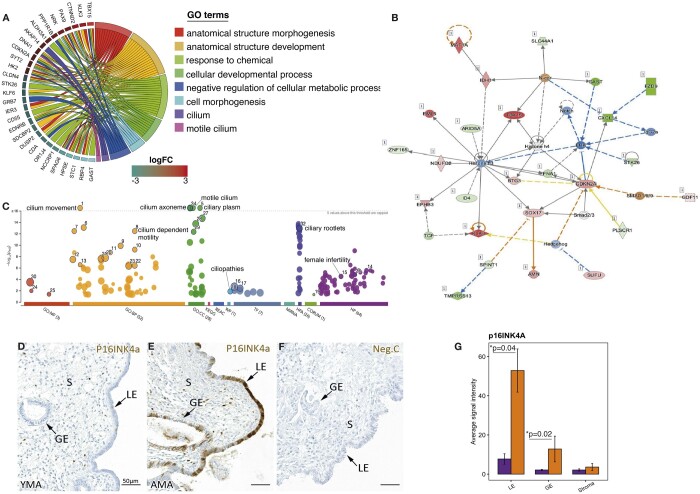
**Genes dysregulated in the endometrium of women of advanced maternal age.** (**A**) The top differentially expressed (DE) genes and their biological functions, FC > 1, Benjamin–Hochberg adjusted *P*-value < 0.05. (**B**) Molecular interaction network generated using Ingenuity pathway analysis (IPA). One of the top significant networks (score 35) suggests the interaction between the p16 (*CDKN2A*) senescence marker with Notch, ERK and Hedgehog signalling. (**C**) Functional analysis of DE genes in advanced maternal age (AMA) samples represent gene ontology (GO) terms significantly enriched in the endometrium of AMA samples compared to young maternal age (YMA) samples. The most significantly enriched terms were cilium (GO: 0005929, *P* = 2.42×10^−20^), cilium movement (GO: 0003341, *P* = 4.74×10^−17^), axoneme (GO: 0005930, *P* = 2.00×10^−17^), and motile cilium (GO: 0031514, *P* = 1.44×10^−17^). BP, biological processes; CC, cellular component; HP, human phenotype; MF, molecular function; REAC, reactome; TF, transcription factor; HPA, Human Protein Atlas; WP, WikiPathways. GO terms marked with numbers are described in Supplementary Table S3A. (**D** and **E**) Immunohistochemistry (IHC) analysis of p16 (p16^INK4a^) senescence protein in YMA and AMA samples, respectively. (**F**) Negative control staining for p16, in which the primary antibody was omitted. (**G**) p16^INK4a^ protein expression in endometrial cellular compartments, identified by IHC from YMA patients and AMA patients. p16 exhibited significantly higher expression in luminal epithelial cells (LE) and glandular epithelial cells (GE) (Wilcoxon rank-sum test *P* < 0.05).

After multiple testing corrections, 491 significant DEGs, 271 upregulated and 220 downregulated, were identified in the endometrium of the AMA group compared to the YMA group (Supplementary Table S2). The changes in the expression rate ranged from a –7 to a 7-fold change. No differences were observed in the expression of steroid hormone receptors between the groups. The genes with the highest expression fold change were *GAST*, *TBX15*, *RBP4*, *GP2*, *ADGRF1*, *STC1*, *S100P*, *HPSE*, *DEPP1*, *KLK3*, *CXCL14*, and *CTNND2*. The most statistically significant DEGs were *ZNF229*, *C2CD4B*, *TBX15*, *SPAG6*, *OR1J4*, *TOR4A*, *CLDN4*, *GRB7*, *C2CD4A*, *RBP4*, *PPP1R1B*, and *RIMKLB* (Fig. 3A, Supplementary Table S2). Real-time quantitative PCR analyses using validation group samples confirmed the expression differences of several DEGs, including *ALDH3A1*, *EML5*, *SPAG6*, *STC1*, *PPP1R1B*, and *TBX15* (Supplementary Fig. S1B) in AMA endometrial samples compared to YMA samples.

Functional analysis of AMA DEGs showed that the most enriched GO terms were general cellular and organismal developmental processes. Significantly enriched cellular components were cilium (GO: 0005929; *P* adj = 2.42×10^−20^), axoneme (GO: 0005930, *P* = 2.00×10^−17^) and motile cilium (GO: 0031514; *P* adj = 1.44×10^−17^), as well as the biological process cilium movement (GO: 0003341, *P* adj = 4.74×10^−17^), as shown in Fig. 3C. Thus, the enrichment analysis identified GO terms associated with cilial development and functioning (Fig. 3C). A full list of significantly enriched terms is presented in Supplementary Table S3A. IPA analysis indicated nine significant molecular networks, including the networks associated with cell cycle progression and cancer, cellular movement (including immune cell movement), endocrine system disorders (including metabolism disorders), cell morphology, and development (Supplementary Table S3B, IPA score > 21, Benjamini and Hochberg *P*-value < 0.05). According to the IPA, DEGs in these networks are directly or indirectly associated with the cell cycle regulator molecule cyclin-dependent kinase 2A (*CDKN2A*), better known as the p16^INK4a/ARF^ locus, which was upregulated in the AMA group (Fig. 3B, Supplementary Table S2). It encodes a cellular senescence marker, which interacts with Sonic hedgehog and Notch pathways (Fig. 3B, Supplementary Table S3B). *CDKN2A* was also the only AMA gene identified by all three databases for age-associated genes.

When examining DEGs for their putative role in endometrial functioning, we found that many genes upregulated in AMA were associated with cell cycle control (*CDKN2A*), insulin signalling (*TMED6*), and endometrial receptivity (*GALNT12*, *TMED6*, *CLDN4*, *CD55*), while downregulated genes were linked with sugar metabolism and inflammation (*C2CD4A*, *C2CD4B*, *NFKB*), cellular movement and invasion (*SPAG6*, *HPSE*, *GRB7*), and ovarian hormone signalling (*STC1*, *GRB7*, *ALDH3A1*). When compared to previously reported genes associated with endometrial receptivity (Hu *et al.*, 2014; Altmäe *et al.*, 2016; Sigurgeirsson *et al.*, 2017; Suhorutshenko *et al.*, 2018), 26 out of 73 transcriptomic endometrial dating genes (marked with an asterisk in Supplementary Fig. S2A) were dysregulated in AMA samples and 18 AMA genes (Supplementary Fig. S2B) were associated with HRT-specific changes in the endometrium.

### The expression of p16^INK4a^ in endometrial tissue

To localize the expression of the cellular aging marker p16^INK4a^ (p16) in AMA/YMA endometria, IHC analysis was performed in four YMA and four AMA endometrial samples. The analysis of protein staining intensity showed that p16 was significantly higher in the luminal and glandular epithelial cells of the AMA samples (*P* = 0.049 and 0.026, respectively, Wilcoxon rank-sum test, two-sided), when compared to the YMA group (Fig. 3D–G). p16 was localized in both the cytoplasm and nuclei of epithelial and stromal cells, which was considered as positive staining. No differences in the cellular localization of p16 were observed between the groups. The staining intensity showed a higher degree of variation in the AMA group than in the YMA group, indicating possible age-related endometrial tissue heterogeneity.

### The expression of AMA genes across the woman’s reproductive lifespan

**Figure 4.**
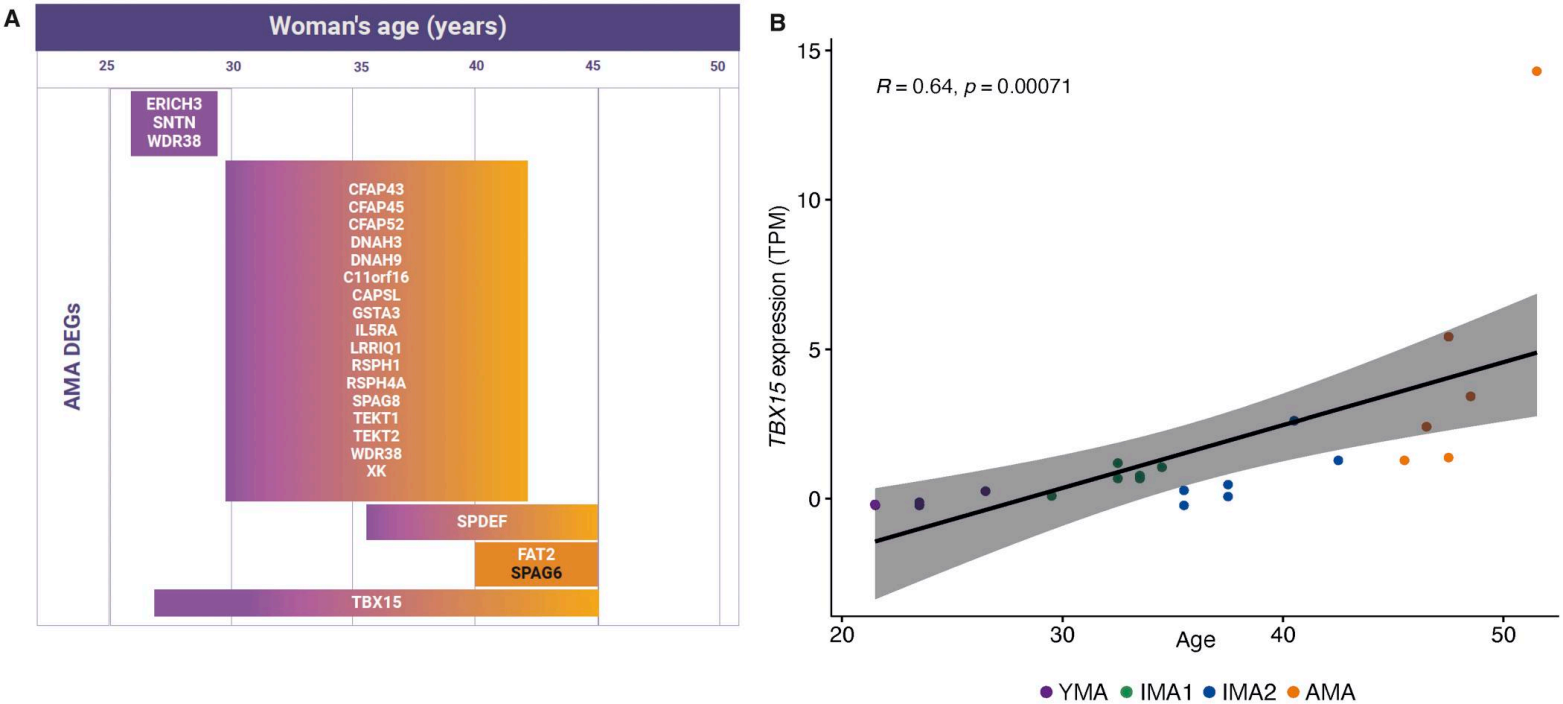
**The expression of advanced age-associated genes in the natural cycles across woman’s reproductive lifespan.** (**A**) The pairwise comparisons among four age groups (young maternal age (YMA), intermediate maternal age groups 1 and 2 (IMA1, IMA2), and advanced maternal age (AMA)) revealed the possible onset of specific AMA-related gene expression changes (Bonferroni adj *P*-value < 0.05). The upregulated genes in groups with higher age are presented in white and the down-regulated genes are presented in black, which corresponded to the direction of gene expression changes in the study group. (**B**) Gene *TBX15*, which participates in the epithelial-to-mesenchymal transition of epithelial cells, showed a positive correlation with age of the women (*R* = 0.64, *P*-value = 0.00071).

The transcriptomes of 24 endometrial samples from women aged between 20 and 50 years obtained in NC were analysed in four age groups (YMA, IMA1, IMA2, and AMA). The expression of AMA genes was pairwise compared among four age groups across a woman’s reproductive lifespan (between 20 and 50 years old). As a result, several AMA genes changed significantly at different ages (Bonferroni adjusted *P* < 0.05). We present these genes in Fig. 4. Most of the age-associated changes occurred in the late thirties and early forties (Fig. 4A). However, the AMA genes *ERICH3*, *SNTN*, and *WDR38* were significant only between the twenties and early thirties (IMA1 vs YMA). The expression of the *TBX15* gene, the most highly differentially expressed gene in the AMA group, changed significantly at several ages and correlated positively with a woman’s age (R = 0.64, *P* = 0.00071) (Fig. 4B). The steroid hormone receptor genes showed no significant changes between groups. The full list of significant DEGs among the four age groups is presented in Supplementary Table S4.

### Endometrial cell population proportions in YMA and AMA groups

**Figure 5.**
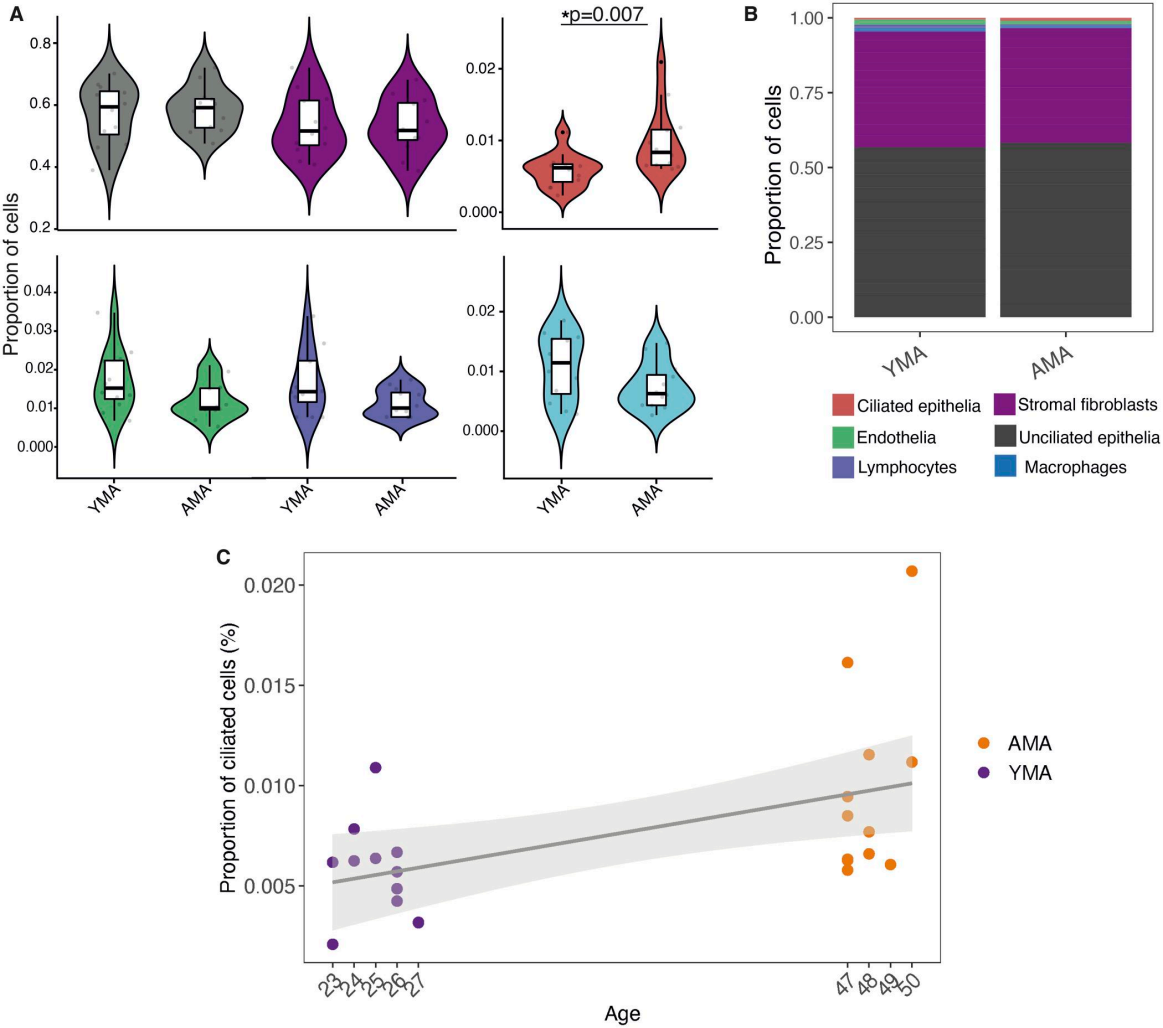
**Cell type proportions per age group determined by deconvolution analysis.** All cell types have an associated colour, shown in the key. (**A**) Violin plots show the changes in cellular proportions between young and advanced maternal age groups for six cell types based on the Fluidigm C1 single-cell RNA-sequencing dataset. The difference in epithelial multiciliated cells (MCCs) between young and advanced maternal age groups was statistically significant, with increased numbers of MCCs in advanced maternal age (AMA) samples, according to two-sided Wilcoxon test with Bonferroni correction, *P* = 0.007. The boxplots show the interquartile range (box limits) and median (centre black line) of cellular proportion levels. A significantly higher proportion of MCCs was observed in the AMA group. (**B**) Stacked bar plots depict percentages of six cell types in young and advanced maternal age groups based on the Fluidigm C1 single-cell RNA-sequencing dataset. (**C**) Proportions of endometrial MCCs aligned according to age of the women.

Histological evaluation of the study group samples according to the Noyes criteria (Noyes *et al.*, 1950) confirmed that all endometrial samples were in the secretory phase and corresponded to the receptive endometrium according to the beREADY algorithm (Meltsov *et al.*, 2023). The results based on the Fluidigm C1 single-cell dataset of six endometrial cell types (Wang *et al.*, 2020) showed a significantly higher proportion of ciliated cells (epithelial MCCs) in AMA samples than in YMA samples (proportion, 0.01 ± 0.005 vs 0.004 ± 0.002; *P* < 0.05, Wilcoxon rank-sum test, two-sided; Fig. 5A and B). The same analysis was also performed with the 10× Genomics single-cell dataset of eight endometrial cell types (Wang *et al.*, 2020). It also revealed that MCCs were more abundant in the AMA samples than in their YMA counterparts (proportion, 0.006 ± 0.009 vs 0.0009 ± 0.002), although the difference was not statistically significant. This could be due to the different sample preparation and analysis techniques used to establish these two single-cell RNA-seq datasets. When the samples were ordered by the age of the women, it became clear that the AMA group samples had a higher, but more variable proportion of the MCCs than the YMA group (Fig. 5C).

### IHC analysis of MCCs in the endometrium

**Figure 6.**
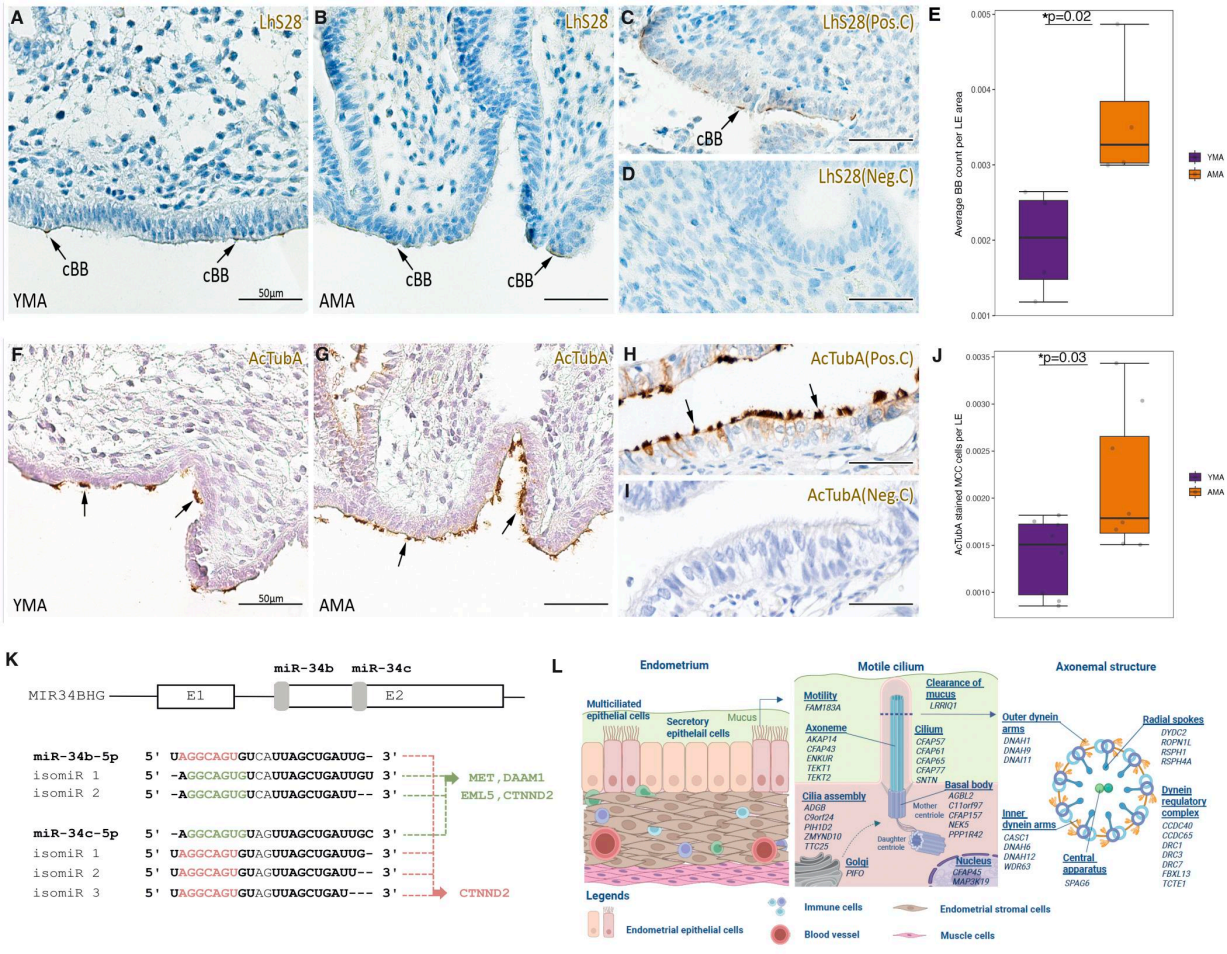
**Analysis of multiciliated cells in endometrial samples from young and advanced maternal age group women.** (**A**–**D**) Basal body staining using LhS28 antibody in endometrial tissue: young maternal age (YMA), advanced maternal age (AMA), Day 16 of natural cycle (positive control of endometrial tissue sample from a fertile YMA woman) and a negative control, respectively. (**E**) Relative basal body (BB) count per luminal epithelium (LE) area, calculated based on LhS28 staining in human endometrium, showed significantly higher numbers of BBs in the AMA group, according to independent sample Student’s *t*-test, *P* = 0.02. (**F**–**I**) Motile cilia staining using an acetylated α-tubulin (AcTubA) antibody in endometrial tissue: YMA, AMA, a fallopian tube sample as a positive control, and a negative control, respectively. Primary antibodies were omitted from negative control experiments. (**J**) Relative multiciliated cells (MCC) count per luminal epithelium (LE) area, calculated based on AcTubA staining in human endometrium, showed significantly higher numbers of MCCs in the AMA group, according to independent sample Student’s *t*-test, *P* = 0.03. (**K**) Genomic localization of differentially expressed miRNAs associated with cilia and the target genes. miR-34b and miR-34c are localized to a single cluster that resides in the host gene MIR34BHG on chromosome 11. The target genes for miR-34b and miR-34c and their isomiRs are predicted with the miRDB database according to their sequence, which is depicted in colour. (**L**) Structure of endometrial multiciliated cells (MCCs) and functions of motile cilia affected by AMA. The gene names represent differentially expressed genes in the AMA endometrium (Benjamin and Hochberg (BH) adjusted *P*-value < 0.05) and are associated with motile cilia. baseMean, average expression in analysed samples; Log2FC, logarithmic expression fold change; padj, BH-adjusted p-value; PMID, PubMed identification number. The figure was created using BioRender.com.

IHC analyses of the cilia BBs and motile cilia, using anti-LhS28 and anti-AcTubA antibodies, respectively, were also conducted to determine whether the AMA samples had more ciliated cells than the YMA samples. The stained basal bodies of the ciliated cells were observed as crescent-shaped organelles near the surface of the epithelial cells with motile cilia visible on the surface (Fig. 6A–D). The number of positively stained BBs was counted in image analysis per area of the LE, i.e. BB counts per LE, in both the YMA and AMA groups. The average BB count per LE area was higher in the AMA group than in the YMA group (3.6 × 10^−3^ (SD ± 8 × 10^−4^) vs 2.0×10^−3^ (SD ± 9×10^−4^), respectively; Unpaired samples Student’s *t*-test, *P* = 0.02) (Fig. 6E). AcTubA-stained cilial structures were seen on the top of MCCs (Fig. 6F–I). The number of MCCs per LE area was higher also in AMA samples (2.16 × 10^−3^ (SD ± 7 × 10^−4^)) than in the YMA group (1.4 × 10^−3^ (SD ± 4 × 10^−4^)) (Unpaired samples Student’s *t*-test, *P* = 0.03) (Fig. 6F–J), which also supported the results of the deconvolution analysis.

### DE of miRNAs in AMA vs YMA endometrium

To provide further evidence for AMA gene involvement in ciliogenesis via epigenetic miRNA interaction, we analysed the profiles of small non-coding miRNAs in 11 YMA and 11 AMA endometrial biopsies using small-RNA-seq performed by a size-based enrichment step with magnetic beads. A total of 220 differentially expressed miR/isomiRs were identified in the AMA group, 91 upregulated and 129 downregulated. The top 30 differentially expressed miRNAs, templated and non-templated isomiRs are presented in Supplementary Fig. S3 A–C. Of these, the cilia-associated miR34 family was represented by two RefSeqs (miR-34b-5p and miR-34c-5p) and 11 isomiRs, which had the highest log_2_FC values (Supplementary Fig. S3C). The miR-34b-5p isoform was most abundantly expressed within the family in AMA endometria. Notably, miR-34b and miR-34c were localized to a single cluster that resides in the host gene MIR34BHG on chromosome 11, as shown in Fig. 6K. Most of the isomiRs had a 5' shift of one nucleotide relative to the corresponding canonical microRNA. This leads to a change in the ‘seed’ regions, while the ‘seed’ of miR-34b matches those of the miR-34c isomiRs and vice versa. Among the predicted target genes, four were differentially expressed in AMA: *MET*, *DAAM1*, *EML5* (targets of miR-34c-5p and the isomiRs of miR-34b-5p), and *CTNND2*, which is a target of all miR34 family members (Fig. 6K, Supplementary Table S2), which regulate motile cilia development (Song *et al.*, 2014; Bonser *et al.*, 2021).

### Cilia-associated genes among AMA DEGs

To understand the impact of endometrial aging on cilial cell structure and function, we compared our DEG list with publicly available datasets. A total of 93 AMA DEGs were identified as highly expressed in the ciliated cells of human fallopian tube and endometrial tissue, based on the Human Protein Atlas (https://www.proteinatlas.org). These genes were then searched manually in PubMed (https://pubmed.ncbi.nlm.nih.gov) for associations with cilia structures and/or functions. We found 62 publications that provided evidence of cilia association for 84 AMA genes. The identified cilia-associated AMA DEGs are presented in Supplementary Table S5, and their association with cilial structures is shown in Fig. 6L.

In the NC validation group, most of the cilia-associated AMA DEGs were significant only between the YMA and AMA groups (Supplementary Fig. S4, Supplementary Table S4), confirming the results from the main study groups (Supplementary Table S5). The scatter plot illustrates the expression of cilia-associated genes at different ages, where the dots represent the samples. The results suggest a lower expression of cilia genes, except for *SPAG6* gene, in the YMA, IMA1, and IMA2 groups, and a higher expression in the AMA group.

## Discussion

This retrospective study delves into the phenomenon of endometrial aging with the goal of comprehending its mechanisms, pinpointing its onset, and uncovering its potential influence on endometrial function. Here, we compared the transcriptome profiles of endometrial biopsies taken during the WOI period from women of young and advanced reproductive ages. Compared to other endometrial aging studies, we analysed participants who followed a similar endometrial preparation protocol that minimizes inter-individual differences due to hormonal status bias and validated our findings using samples from NC. As HRT is often administered to AMA patients preparing for FET, our study provides considerable evidence of the impact of age on the development of endometrial tissue, as well as its molecular profile and cellular composition in women undergoing ART.

To begin with, the analysis of endometrial receptivity reports of women undergoing HRT showed that the endometrium of women over the age of 45 has a lower receptivity potential than that of women in their twenties (Fig. 2), which prompted us to investigate this phenomenon further. The comparison of gene expression profiles of YMA and AMA endometria revealed a set of genes that were dysregulated in the older women (Supplementary Table S2). The pathways most affected by age were cellular remodelling, cilial movement and cell migration, and immune response, whereas most enriched GO terms were associated with motile cilia (Fig. 3C, Supplementary Table S3), arising from a high proportion of cilia-associated genes (17.1%; 84 out of 491) (Supplementary Table S5), which aligns with previous work by the Díaz-Gimeno group (Devesa-Peiro *et al.*, 2022). Although most genes change significantly during the forties, early alterations in *SNTN* and *WDR38* in epithelial cells may signify the early onset of changes in the MCCs (Fig. 4A, Supplementary Fig. S4, Supplementary Table S4). The deconvolution analysis using cell type-specific gene expression profiles showed a significantly higher proportion of MCCs in the AMA group, confirmed by IHC (Figs 5A–C and 6). Previous studies have shown that MCCs are a distinct cell subtype within the endometrial epithelium, characterized by a specific transcriptomic profile (Wang *et al.*, 2020; Garcia-Alonso *et al.*, 2021). Motile cilia are microtubule-based cellular structures having a 9 + 2 axonemal organization and accessory structures that enable cilia movement. In the female reproductive tract, MCCs guarantee the movement of mucus and the embryo along the epithelial surface. Ciliogenesis is driven, although not exclusively, by oestrogen and through the inhibition of Notch signalling (Haider *et al.*, 2019), and regulated by miR34/449 family microRNAs (Marcet *et al.*, 2011; Song *et al.*, 2014; Chevalier *et al.*, 2015). Our miRNA analysis also confirmed the upregulation of cilia-regulating miRNAs and their target genes in AMA women (Fig. 6K, Supplementary Fig. S3).

During the menstrual cycle, rising oestrogen levels typically promote MCC proliferation and differentiation from epithelial progenitor cells, peaking around cycle Days 15–16. At this point, progesterone produced by the corpus luteum opposes oestrogen action, reducing the number of MCCs by the time of potential implantation (Masterton *et al.*, 1975; More and Masterton, 1976). These cyclic events highlight the importance of appropriate cellular proportions of the MCCs in ensuing endometrial receptivity, including both sufficient proliferation and a programmed decrease in MCC number. The observed shift in MCC proportion may be caused by the increased production of ciliated cells or their insufficient decrease under the influence of secretory phase hormones in AMA women. This suggests that both an excess of oestrogen and a lack of progesterone activity may produce a larger amount of MCCs. Considering that MCCs are the major epithelial cell type in fallopian tubes (Lyons *et al.*, 2006), their higher abundance might not favour embryo implantation in the uterus.

Exploring AMA DEGs, we identified a higher expression of *CDKN2A* (Fig. 3B and G, Supplementary Table S2), which encodes cyclin-dependent kinase (e.g. p16^INK4a^), a common biomarker of aging (Martin *et al.*, 2014). Both the cytoplasmic and nuclear staining observed conformed with the endometrial cellular localization of p16 previously described by Parvanov *et al.* (2021). While nuclear overexpression is associated with pathological conditions driven by p16 activation, cytoplasmic p16 accumulation was suggested to be the sign of inactive p16 interacting with other proteins that prevent its activity (Martin *et al.*, 2014; Mendaza *et al*., 2020; Parvanov *et al.*, 2021). Although it has been suggested that p16-positive epithelial cells are required for a supportive environment at the WOI, it has also been shown that p16-staining in endometrial epithelial cells correlates positively with a woman’s age (Parvanov *et al.*, 2021). Our molecular network analysis revealed the interaction of p16 with Notch signalling, which also regulates cell cycle progression (Fig. 3B). Normally, activated Notch induces apoptosis in MCCs in vertebrate animals (Tasca *et al.*, 2021). In endometrium, senescent cells were shown to trigger overexpression of p16 by activating the mechanism of premature senescence (Vassilieva *et al.*, 2018). Notably, the accumulation of putatively inactive p16 in the cytoplasm has also been associated with aging (Idda *et al.*, 2020). Although it is unclear how p16 accumulation affects the human endometrial epithelium, it has been shown that p16 overexpression is associated with endometrial tubal metaplasia, characterized by a higher number of MCCs in the endometrium, and is considered a specific marker for dysplastic and neoplastic epithelial cells (Klaes *et al.*, 2001; Horree *et al.*, 2007). The detailed role of p16 in the development and function of endometrial MCCs remains largely understudied. This may be due to the fact that changes in CDKN2A/p16 may remain sub-significant in NC endometrium, as confirmed by the study of validation group samples (Supplementary Table S4).

Our results suggest that the expression of several genes may impact endometrial aging. For example, *TBX15*, the most highly upregulated gene in the AMA group, may play an important role in endometrial aging as its expression consistently increased during a woman’s reproductive lifespan (Figs 3B and 4A and B, Supplementary Table S2 and S4). This gene is expressed by epithelial cells and associated with epithelial-to-mesenchymal transition (EMT) (Niu *et al.*, 2022). The upregulation of *TBX15* reduces apoptosis through a nuclear factor kappa-light-chain-enhancer of activated B cells (NF-κB)-dependent mechanism (Arribas *et al.*, 2016). The EMT is normally induced by embryonic signals, enabling endometrial luminal cells to acquire the characteristics of motile epithelial cells (Owusu-Akyaw *et al.*, 2019); however, its strong activation in the absence of embryos observed in AMA samples may compromise normal epithelial function. We also identified a set of DEGs previously described in association with endometrial receptivity, such as *CDA*, *CD55*, *CLDN4*, *GAST LAMB3*, and *STC1* (Haouzi *et al.*, 2008; Díaz-Gimeno *et al.*, 2011; Altmäe *et al.*, 2017; Enciso *et al.*, 2018) (Fig. 3A, Supplementary Table S2, Supplementary Fig. S2). The disturbance of oestrogen signalling in AMA is supported by the changes in direct oestrogen-responsive genes, such as *GRB7*, *CD55*, and *CLDN4*. Although synthetic progesterone is administered to activate progesterone receptors in the endometrium during HRT, and no differences in progesterone receptor expression were revealed between the groups, a higher proportion of MCCs together with the observed downregulation of some progesterone-regulated genes (e.g. *CLDN4*, *ARID5B*, *STC1*, and *C2CD4B*) might indicate suboptimal progesterone action in the AMA endometrium (Supplementary Fig. S2). It is unclear whether aging affects the way progesterone acts in the human endometrium, but animal studies suggest the possible effect of aging on progesterone sensitivity (Li *et al.*, 2017).

We have to acknowledge some limitations of our study. While it provides strong evidence that age affects MCC differentiation in the AMA endometrium, this is a retrospective study, and our clinical data are limited. Blood hormone levels were not measured for all participants, and the endometrial pathology data may not be entirely comprehensive. Nevertheless, our study provides considerable evidence that age affects the development of the endometrial epithelium and its functions. The current study is the first to show that older women have a significantly higher proportion of ciliated epithelial cells in their endometrium. The development of epithelial MCCs is strongly associated with cell cycle regulation and can be altered by cellular senescence. Here, we propose that the accumulation of senescent epithelial cells might lead to a shift in the proportions of epithelial cell subpopulations, indicating an increased age-conferred risk for metaplasia in AMA women. However, it remains unclear whether this shift alone can cause implantation failure or whether it exacerbates other factors that compromise endometrial receptivity. Our study also provides evidence that aging does not occur at a certain time point and is specific to every individual. Therefore, a personalized strategy is required to address endometrial aging in clinical practice and the findings of this study contribute significantly to the advancement of fundamental knowledge in this area.

## Supplementary Material

hoae048_Supplementary_Data

## Data Availability

FASTQ files for bulk RNA-seq and raw count matrices were deposited at the Gene Expression Omnibus under accession code GSE236128. All codes used for the analysis are available on GitHub (https://github.com/darinaobukhova/endo_ageing).
