# Supplementary material for: Aging promotes accumulation of senescent and multiciliated cells in human endometrial epithelium
Source: Hum Reprod Open. 2024 Aug 12;2024(3):hoae048. doi: 10.1093/hropen/hoae048 (PMC11344589; doi:10.1093/hropen/hoae048)
Supplement: hoae048_Supplementary_Data [file hoae048_supplementary_data.zip › Supplementary_Fig.S2.pdf]

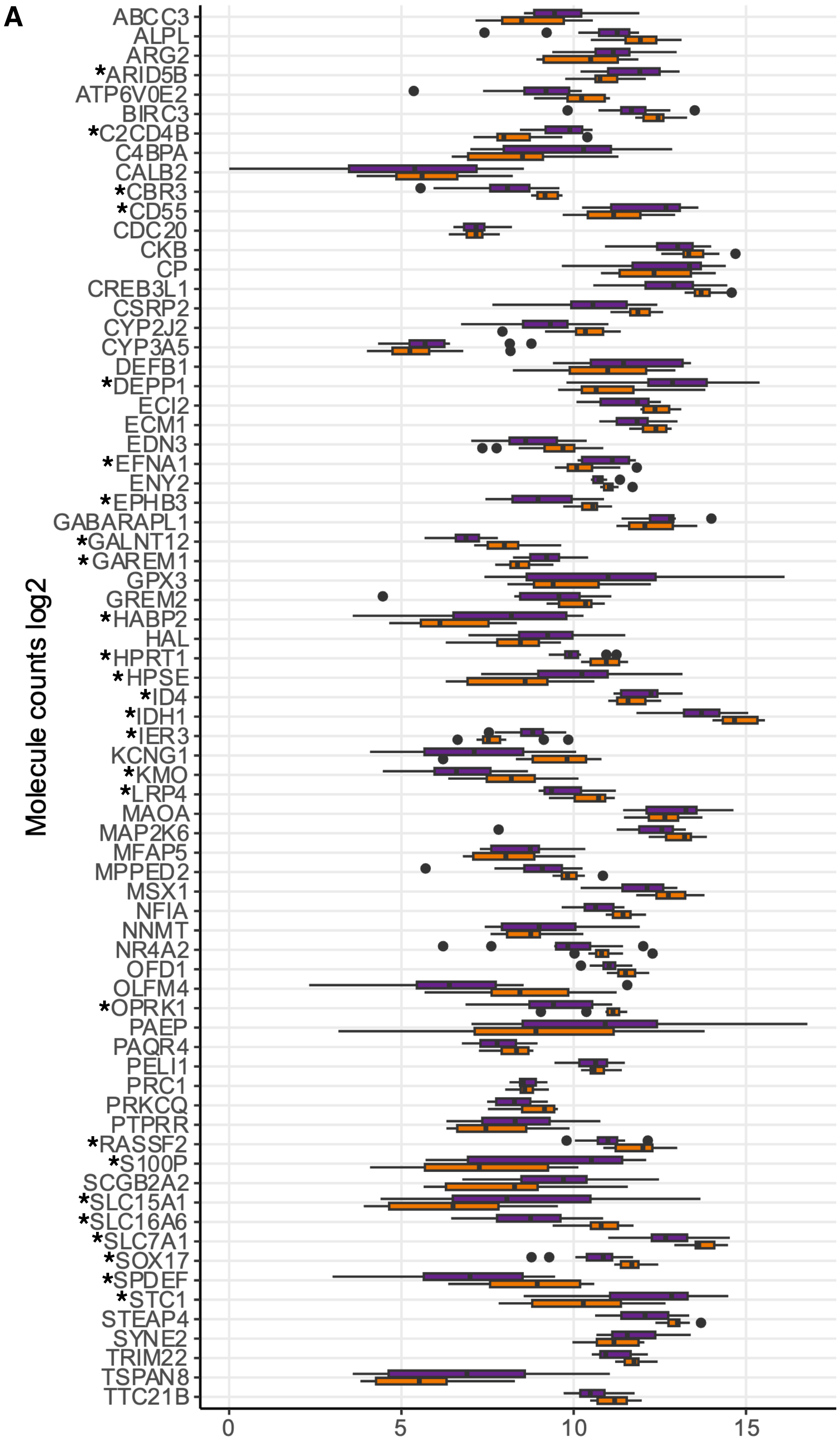


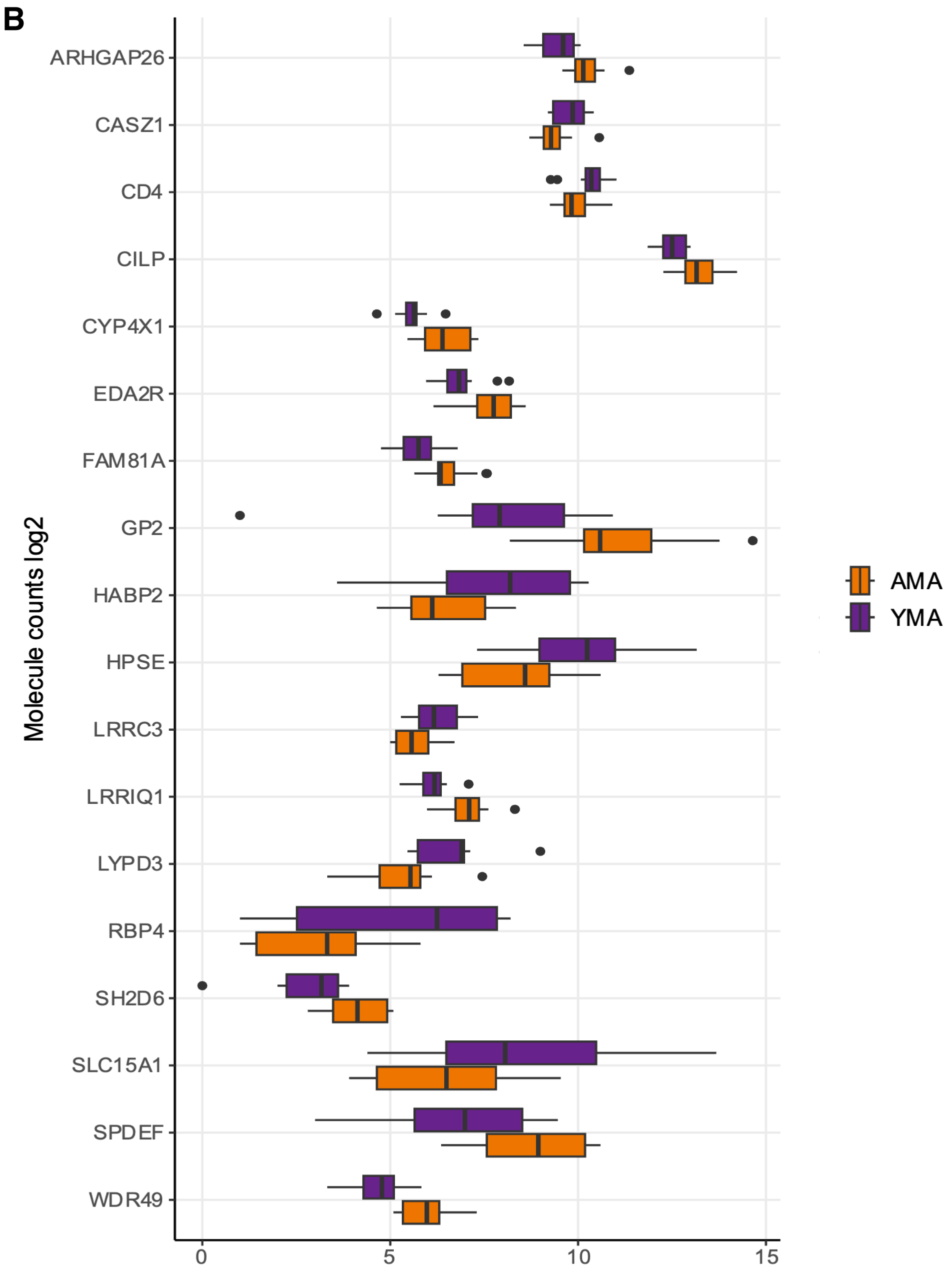


**Supplementary Fig. S2. Receptivity-associated genes in natural cycle and hormone replacement therapy (HRT), differentially expressed in advanced maternal age (AMA) samples.**

(A) Boxplots of 72 AMA significant genes associated with the window of implantation (WOI) in a natural cycle in RNA-seq-based publications. Significant AMA genes are marked with an asterisk.

(B) Boxplots of 18 AMA significant genes associated with HRT.
