# Supplementary material for: Aging promotes accumulation of senescent and multiciliated cells in human endometrial epithelium
Source: Hum Reprod Open. 2024 Aug 12;2024(3):hoae048. doi: 10.1093/hropen/hoae048 (PMC11344589; doi:10.1093/hropen/hoae048)
Supplement: hoae048_Supplementary_Data [file hoae048_supplementary_data.zip › Supplementary_Fig.S3.pdf]

A

Differential expression of miRNAs and isomiRs

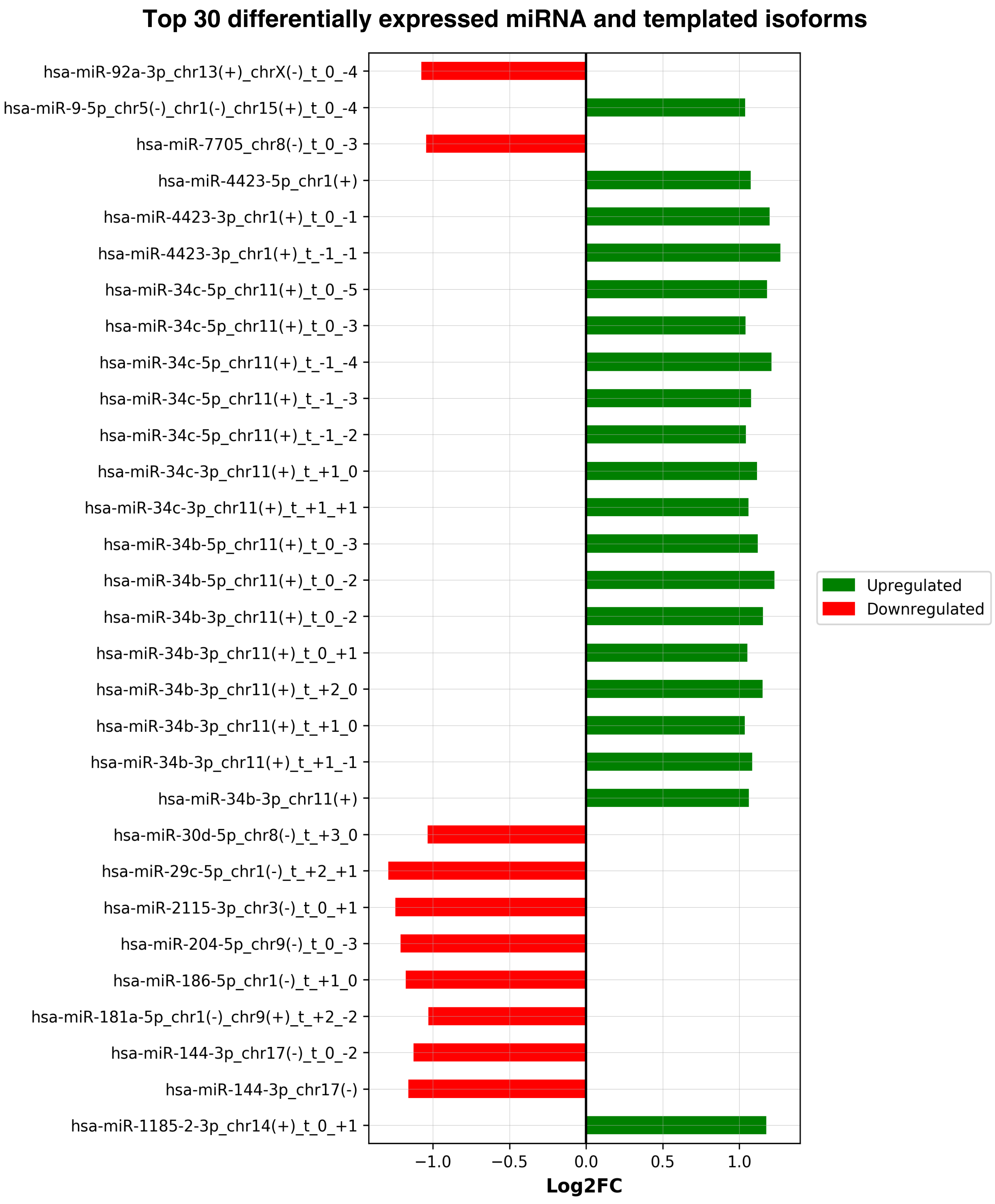

B

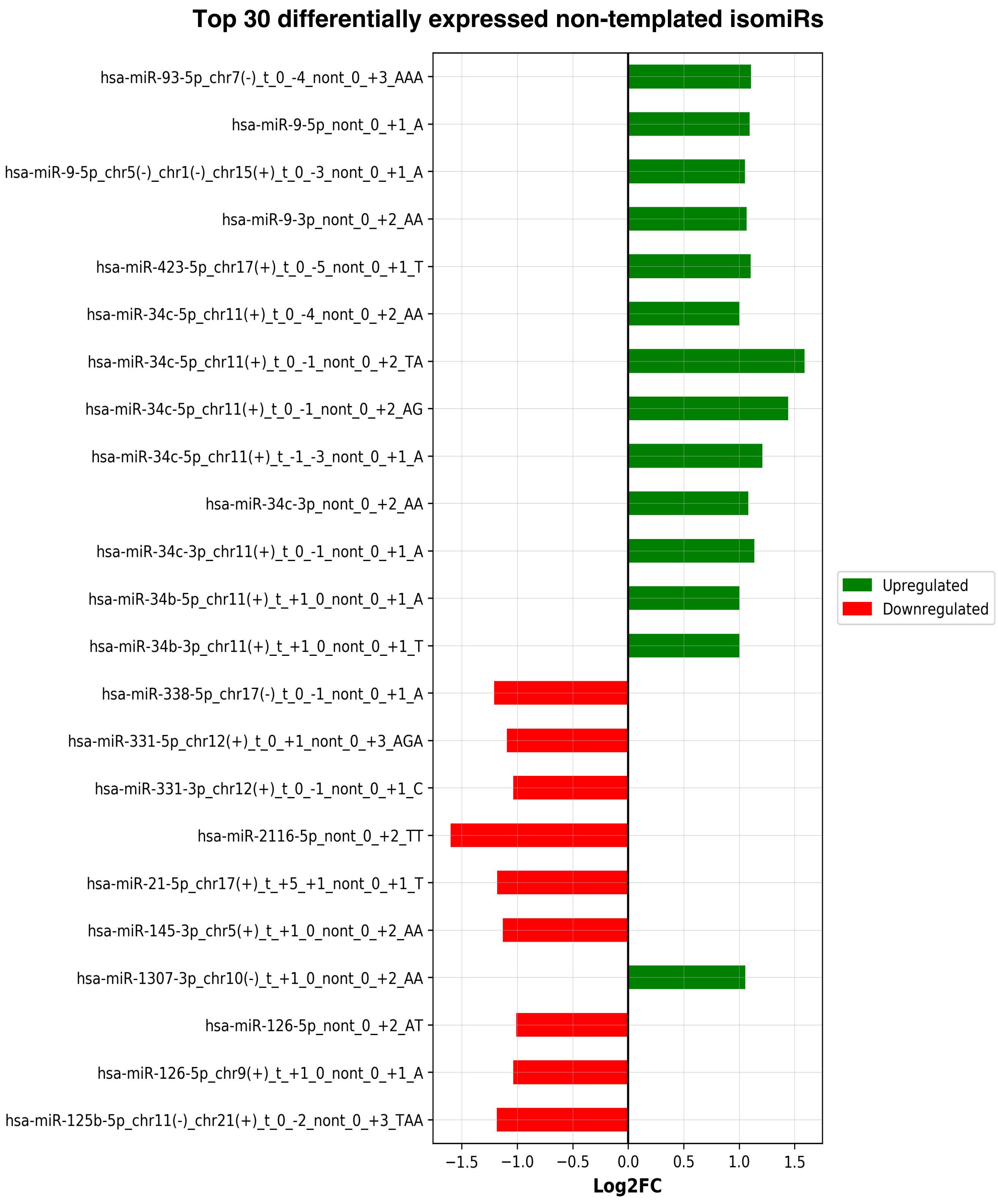

Top 30 differentially expressed non-templated isomiRs
hsa-miR-93-5p_chr7(-)_t_0_-4_nont_0_+3_AAA
hsa-miR-9-5p_nont_0_+1_A
hsa-miR-9-5p_chr5(-)_chr1(-)_chr15(+)_t_0_-3_nont_0_+1_A
hsa-miR-9-3p_nont_0_+2_AA
hsa-miR-423-5p_chr17(+)_t_0_-5_nont_0_+1_T
hsa-miR-34c-5p_chr11(+)_t_0_-4_nont_0_+2_AA
hsa-miR-34c-5p_chr11(+)_t_0_-1_nont_0_+2_TA
hsa-miR-34c-5p_chr11(+)_t_0_-1_nont_0_+2_AG
hsa-miR-34c-5p_chr11(+)_t_-1_-3_nont_0_+1_A
hsa-miR-34c-3p_nont_0_+2_AA
hsa-miR-34c-3p_chr11(+)_t_0_-1_nont_0_+1_A
hsa-miR-34b-5p_chr11(+)_t_+1_0_nont_0_+1_A
hsa-miR-34b-3p_chr11(+)_t_+1_0_nont_0_+1_T
hsa-miR-338-5p_chr17(-)_t_0_-1_nont_0_+1_A
hsa-miR-331-5p_chr12(+)_t_0_+1_nont_0_+3_AGA
hsa-miR-331-3p_chr12(+)_t_0_-1_nont_0_+1_C
hsa-miR-2116-5p_nont_0_+2_TT
hsa-miR-21-5p_chr17(+)_t_+5_+1_nont_0_+1_T
hsa-miR-145-3p_chr5(+)_t_+1_0_nont_0_+2_AA
hsa-miR-1307-3p_chr10(-)_t_+1_0_nont_0_+2_AA
hsa-miR-126-5p_nont_0_+2_AT
hsa-miR-126-5p_chr9(+)_t_+1_0_nont_0_+1_A
hsa-miR-125b-5p_chr11(-)_chr21(+)_t_0_-2_nont_0_+3_TAA
−1.5
−1.0
−0.5
0.0
0.5
1.0
1.5
Log2FC
Upregulated
Downregulated

C

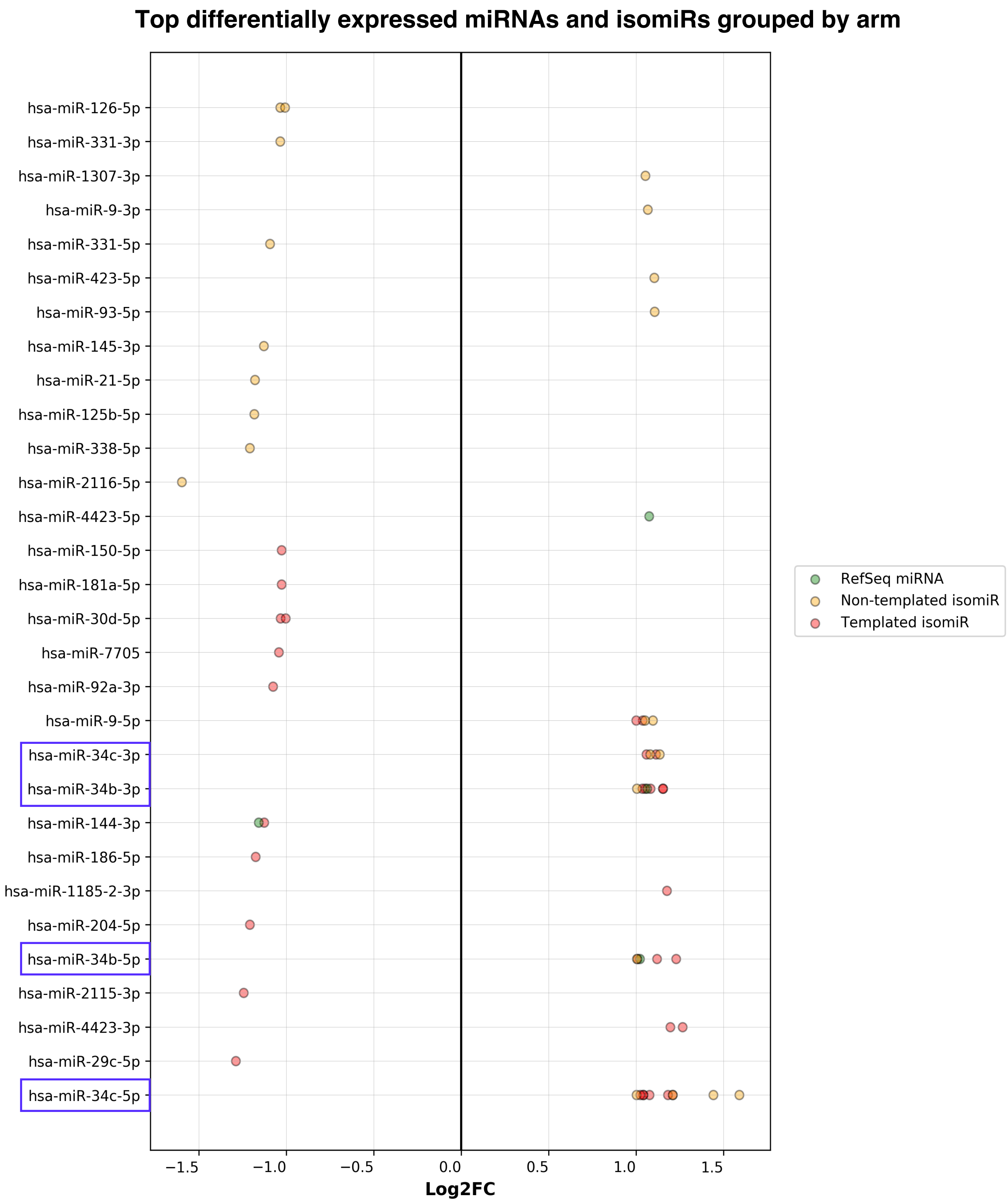

Supplementary Fig. S3. Significant miRNAs and isomiRs, differentially expressed in advanced maternal age (AMA) samples compared to young maternal age (YMA) group ($p < 0.05$, non-adjusted).
(A) Top 30 differentially expressed miRNA and templated isoforms.
(B) Top 30 differentially expressed non-templated isomiRs.
(C) Top differentially expressed miRNAs and isomiRs grouped by miRNA arm. Logarithmic fold change of miRNA expression (log2FC) is shown as positive where miRNA is upregulated in AMA group, and negative where miRNA is downregulated, in relation to YMA. Cilial development-associated miRNAs are presented in boxes.
