# Supplementary material for: Aging promotes accumulation of senescent and multiciliated cells in human endometrial epithelium
Source: Hum Reprod Open. 2024 Aug 12;2024(3):hoae048. doi: 10.1093/hropen/hoae048 (PMC11344589; doi:10.1093/hropen/hoae048)
Supplement: hoae048_Supplementary_Data [file hoae048_supplementary_data.zip › Supplementary_Fig.S4.pdf]

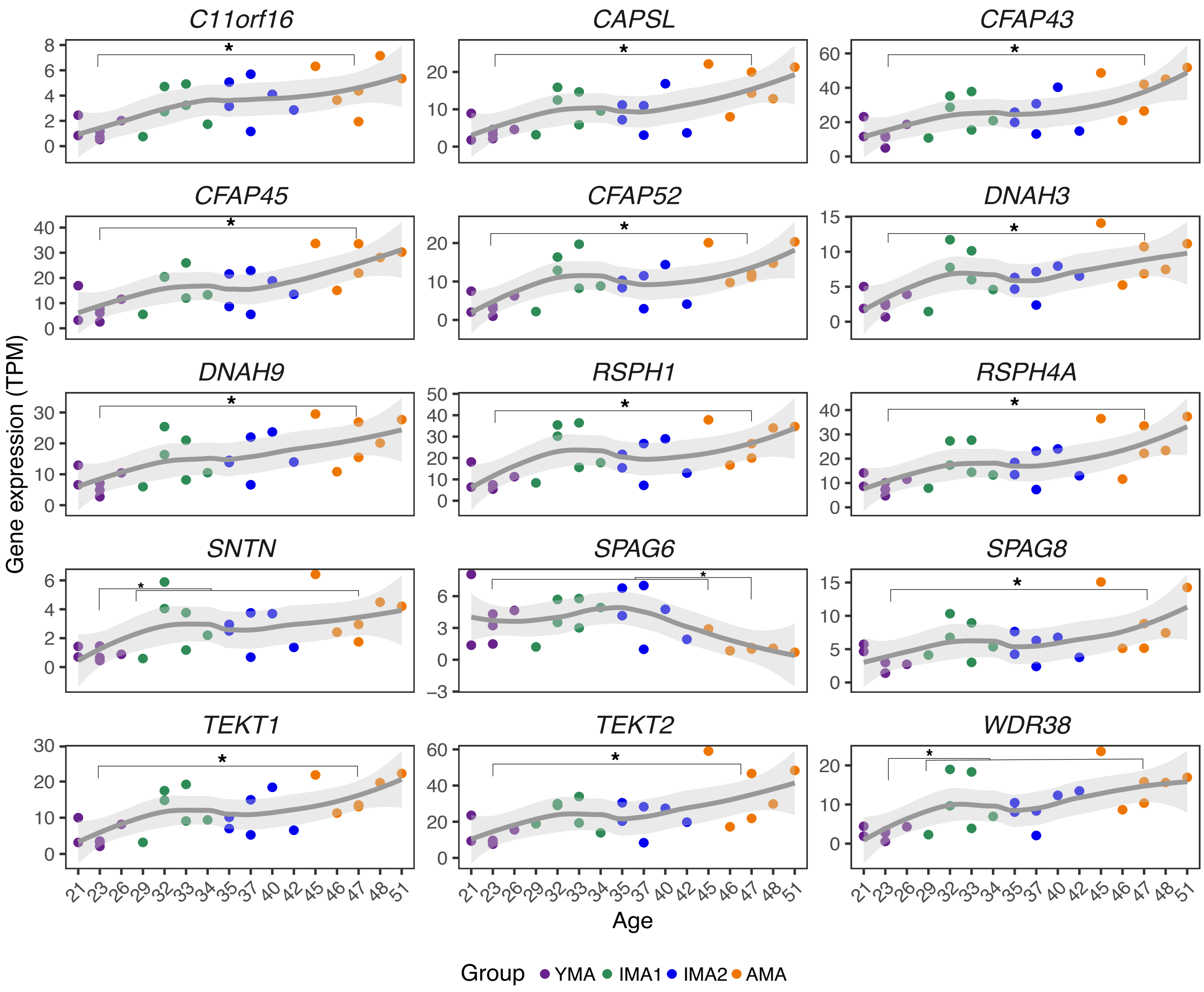

Supplementary Fig. S4. The expression changes of cilia-associated genes in natural cycles during woman's reproductive lifespan.

15 cilia-associated AMA genes showed significant gene expression change between YMA and AMA groups in natural menstrual cycles, Bonferroni adjusted $p < 0.05$.
