# Supplementary material for: Aging promotes accumulation of senescent and multiciliated cells in human endometrial epithelium
Source: Hum Reprod Open. 2024 Aug 12;2024(3):hoae048. doi: 10.1093/hropen/hoae048 (PMC11344589; doi:10.1093/hropen/hoae048)
Supplement: hoae048_Supplementary_Data [file hoae048_supplementary_data.zip › HRO-23-0309-R1-SuppFileS1.docx]

**Supplementary File S1**.

**Detailed description of bioinformatic analyses performed in the study**

Reads were mapped to the *Homo sapiens* reference genome (UCSC release GRCh37/hg19), using *STAR* (v. 2.7.9) in “Basic” two-pass mode and in “GeneCounts” quantification mode*.* FastQC (v. 0.11.9) and SAMtools (v. 1.14) were used to extract the quality control metrics. All the samples had more than 20M reads aligned to the genome, with an alignment percentage of >85%. The quality control metrics and alignment statistics were summarised in an HTML report using MultiQC (v. 1.12). The mapped reads were estimated at the gene level using the Ensembl database for annotation. The raw expression count matrix was produced using *FeatureCounts* (v. 2.0.1). The batch effect of the sequencing run was corrected for using the ‘Combat-Seq2’ method from the *sva* R package (v. 3.42.0), as it uses a negative binomial regression model, suitable for modelling the characteristics of bulk RNA-seq count data. Subsequently, genes in which the number of mapped reads was < 5 in more than 75% of samples were excluded from further analysis.

**Detection and validation of differentially expressed genes (DEGs)**

To identify the genes that are differentially expressed between the AMA and YMA groups, the matrix of batch effect-adjusted counts was supplied to *DESeq2* (v. 1.34.0), using, by default, a filter criterion of Benjamin and Hochberg adjusted p-value < 0.05. The receptivity score was used as a covariate in the design matrix. Since the expected variance of RNA-seq counts increases with the mean, the ‘variance stabilising transformation’ (VST) function from *DESeq2* was used to generate a matrix of transformed counts for sample visualisation. To check endometrial gene expression in relation to age, the top 20 most significant genes were sorted by log_2_FC and clustered hierarchically using the VST-modified counts. This was plotted in heatmaps with accompanying dendrograms using the *pheatmap* package in R (v. 1.0.12).

Following the differential expression analyses, power estimation for RNA-seq experiments was used to determine the appropriate sample size required to achieve the desired level of statistical power. This ensures that the experiment is adequately designed to detect differential expression between two groups, while minimising the risk of both false positive and negative results. Performing power analysis for RNA-seq experiments is challenging due to the complex nature of the data and the methods used. Thus, analytical solutions for power calculations in RNA-seq experiments are not feasible. Instead, the PROspective Power Evaluation for RNA-seq (PROPER) R package provided a simulation-based method to compute power-sample size relationships. With this method, it was computed that given the sample size of 12, the significance level (p adj) of 0.05 and the effect size (log_2_FC) of 0.3, the power estimate was 85%, whereas for the genes with log_2_FC of at least 1, the power was 99%. Thus, the current study design was considered eligible, with well-powered sample sizes available of 12 YMA and 12 AMA women in the study groups.

**RNA sequencing analysis of young, intermediate and advanced maternal age samples from natural cycles**

After the sequencing, read quality and alignment were processed, checked with FastQC (v. 0.11.8) and the reads were aligned to the *Homo sapiens* reference genome (UCSC release GRCh37/hg19), using *STAR* (v. 2.7.9) in “Basic” two-pass mode and in “GeneCounts” quantification mode*.* The raw expression count matrix was produced using *FeatureCounts* (v. 2.0.1). Since the samples were sequenced in 4 batches, the batch effect of the sequencing run was corrected for using the ‘Combat-Seq2’ method from the *sva* R package (v. 3.42.0). Differential expression between four age groups was studied using *DESeq2* (v. 1.34.0). Genes with raw count values lower than 4 in over 75% of samples were excluded from the analysis. To compare differentially expressed genes among four age groups in a pairwise manner, we used “~Age_Group” as the value for the “design” argument in the *DESeqDataSetFromMatrix()* function. Each age group was taken as a reference level to be compared against using the *relevel()* function. To compare YMA and AMA groups with all other groups, “~0 + Age_Group” was used as the value for the “design” argument in the *DESeqDataSetFromMatrix()* function to fit a separate coefficient for each group. We assigned “c(3/4, -1/4)” as a value for the “listValues” argument in the *results()* function, which compares gene expression in the YMA and AMA groups, respectively, against the average of the other age groups.
