# Supplementary material for: Aging promotes accumulation of senescent and multiciliated cells in human endometrial epithelium
Source: Hum Reprod Open. 2024 Aug 12;2024(3):hoae048. doi: 10.1093/hropen/hoae048 (PMC11344589; doi:10.1093/hropen/hoae048)
Supplement: hoae048_Supplementary_Data [file hoae048_supplementary_data.zip › Supplementary_Fig.S1.pdf]

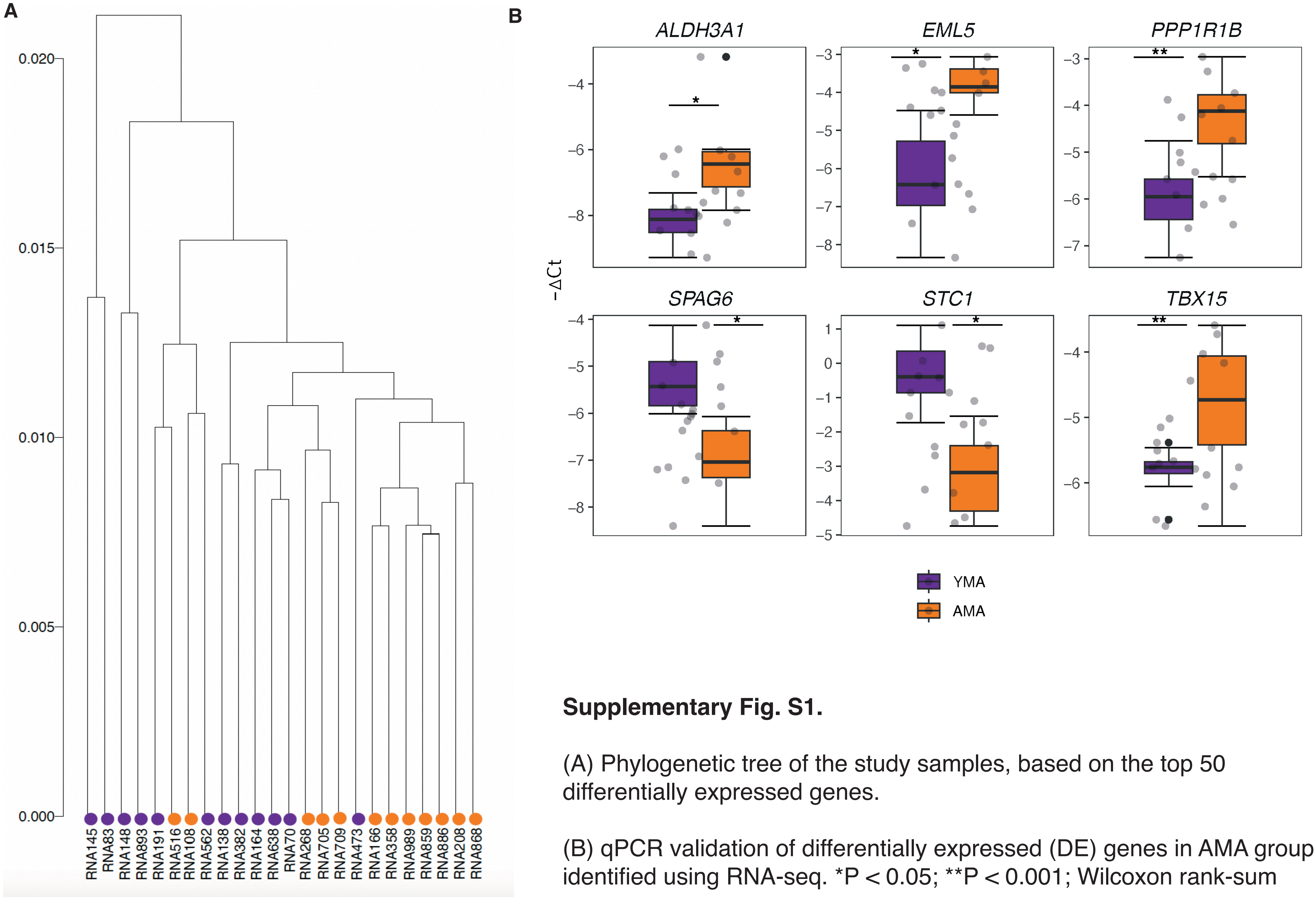

Supplementary Fig. S1.

(A) Phylogenetic tree of the study samples, based on the top 50 differentially expressed genes.

(B) qPCR validation of differentially expressed (DE) genes in AMA group identified using RNA-seq. *$P < 0.05$; **$P < 0.001$; Wilcoxon rank-sum
